# Considerations for homology-based DNA repair in mosquitoes: Impact of sequence heterology and donor template source

**DOI:** 10.1371/journal.pgen.1010060

**Published:** 2022-02-18

**Authors:** Joshua Xin De Ang, Katherine Nevard, Rebekah Ireland, Deepak-Kumar Purusothaman, Sebald A. N. Verkuijl, Lewis Shackleford, Estela Gonzalez, Michelle A. E. Anderson, Luke Alphey

**Affiliations:** 1 The Pirbright Institute, Pirbright, Woking, United Kingdom; 2 Mathematical Ecology Research Group, Department of Zoology, University of Oxford, Oxford, United Kingdom; University of Kentucky, UNITED STATES

## Abstract

The increasing prevalence of insecticide resistance and the ongoing global burden of vector-borne diseases have encouraged new efforts in mosquito control. For *Aedes aegypti*, the most important arboviral vector, integration rates achieved in Cas9-based knock-ins so far have been rather low, highlighting the need to understand gene conversion patterns and other factors that influence homology-directed repair (HDR) events in this species. In this study, we report the effects of sequence mismatches or donor template forms on integration rates. We found that modest sequence differences between construct homology arms [DNA sequence in the donor template which resembles the region flanking the target cut] and genomic target comprising 1.2% nucleotide dissimilarity (heterology) significantly reduced integration rates. While most integrations (59–88%) from plasmid templates were the result of canonical [on target, perfect repair] HDR events, no canonical events were identified from other donor types (i.e. ssDNA, biotinylated ds/ssDNA). Sequencing of the transgene flanking region in 69 individuals with canonical integrations revealed 60% of conversion tracts to be unidirectional and extend up to 220 bp proximal to the break, though in three individuals bidirectional conversion of up to 725 bp was observed.

## Author summary

The field of genetic control of mosquito vectors has progressed rapidly in recent years, especially in Cas9-based control systems, due to its robustness to elicit a species-specific and dispersive control of mosquito population. To generate a Cas9-based integration, Cas9 and sgRNA are used to cleave a chromosomal locus while a plasmid DNA donor, containing a genetic cargo flanked by sequences homologous to the chromosomal locus, is supplied as a repair template. This results in the cargo being copied into the genome through HDR. This form of integration, however, is currently one of the major bottlenecks for researchers as it involves a laborious process of microinjecting mosquito embryos and has rather low integration rates. In this study, we assessed the effects of homologous sequence mismatches and various donor template forms (i.e. plasmid, ssDNA, biotinylated ds/ssDNA) on HDR. We found that sequence mismatches and non-plasmid donors reduced the efficiency and integrity of integration, respectively. By analysing the direction and length of homologous sequence that was copied into the genome concurrently with the cargo, we inferred the mechanism responsible for the integrations observed in our study. These findings will be useful to guide future construct designs for optimal HDR rates in mosquitoes.

## Introduction

*Aedes aegypti* mosquitoes are the primary vector of the viruses that cause dengue [1], chikungunya [2], yellow fever [3] and Zika [4], which account for hundreds of millions of infections each year [5]. Mosquito control programmes aiming to reduce the spread of these diseases largely rely on insecticides. However, issues of resistance [6–8], and an ongoing global disease burden [9], have led to increasing interest in genetic control strategies. Broadly, mosquitoes could be modified to carry a trait which causes refractoriness to a pathogen (population modification strategy) or one that reduces viability or fertility (population suppression strategy).

Researchers have long used transposable elements to insert genes of interest into the genomes of organisms in a pseudo-random, non-site-specific manner [10–12]. The ability to make a double-stranded break (DSB) at a specific genomic locus with CRISPR/Cas9 has more recently enabled site-specific, heritable genetic engineering in mosquitoes and many other organisms [13–18]. Once a DSB is induced, the cell will either repair the break via non-homologous end-joining (NHEJ) which is error-prone, or via homology-directed repair (HDR), copying the sequences of an uncut homologous chromosome. This HDR pathway is exploited to generate transgenic organisms by providing a donor template containing the transgene flanked by “homology arms”–DNA sequences identical to those flanking the expected cut site—usually in the form of a circular or linear dsDNA. Due to the homology arms, the broken strands of the chromosome recognise the donor as a repair template and the transgene is copied as a result of HDR, thereby integrating the injected template sequence into the genome. The use of site-specific knock-ins in *Ae*. *aegypti* have included the development of Cas9-based gene drive systems [19,20], driver lines to express transcriptional activators in specific cell types [21–23], and the study of the effects of amino acid changes on protein function [24]. In other organisms, this tool has been used to tag and study endogenous expression of gene products [25].

Despite the versatility of this tool for both basic and applied research, little is known about the biological processes which take place during HDR events in mosquitoes, and the factors which affect the efficiency of HDR. Previous studies in mammalian cells and *Drosophila melanogaster* have shown that HDR is highly sensitive to sequence heterology (dissimilarity) between the homology arms of the cut chromosome and the donor template and also that gene conversion was largely unidirectional, i.e. occurs only on one end of the DSB [26–28]. Various studies have also shown that HDR rates could be improved by restricting nuclease activity to the S/G2 phase of a cell cycle when HDR is most active, by inhibiting the end-joining pathway, or by modifying/optimising repair templates [13,14,29,30]. However, few such studies have been carried out in mosquitoes and their conclusions may not necessarily be applicable to *Ae*. *aegypti*.

With the increasing need to generate and optimise new transgenic strains for disease control, better understanding of gene conversion and factors affecting HDR in *Ae*. *aegypti* will be valuable to inform future knock-in construct designs. For this reason, we designed constructs with homology arms of varying heterology, and used them as HDR templates in *Ae*. *aegypti* to study the patterns of gene conversion tracts which co-occur with the integration of transgenes and the effects of sequence heterology on HDR efficiency. We also explored the possibility of improving HDR efficiency by using different repair templates (i.e. ssDNA, biotinylated dsDNA, or biotinylated ssDNA) and/or Cas9 (i.e. protein or mRNA fused to a monomeric streptavidin) forms, which have variously been shown to provide benefits in other systems [14,31,32]. We used as a target the *Act4* gene, which has been proposed as a potential target for population suppression homing-based gene drive systems [24,33].

## Results

### Multiplex constructs exhibit reduced integration rates compared to singleplex construct

**Fig 1:**
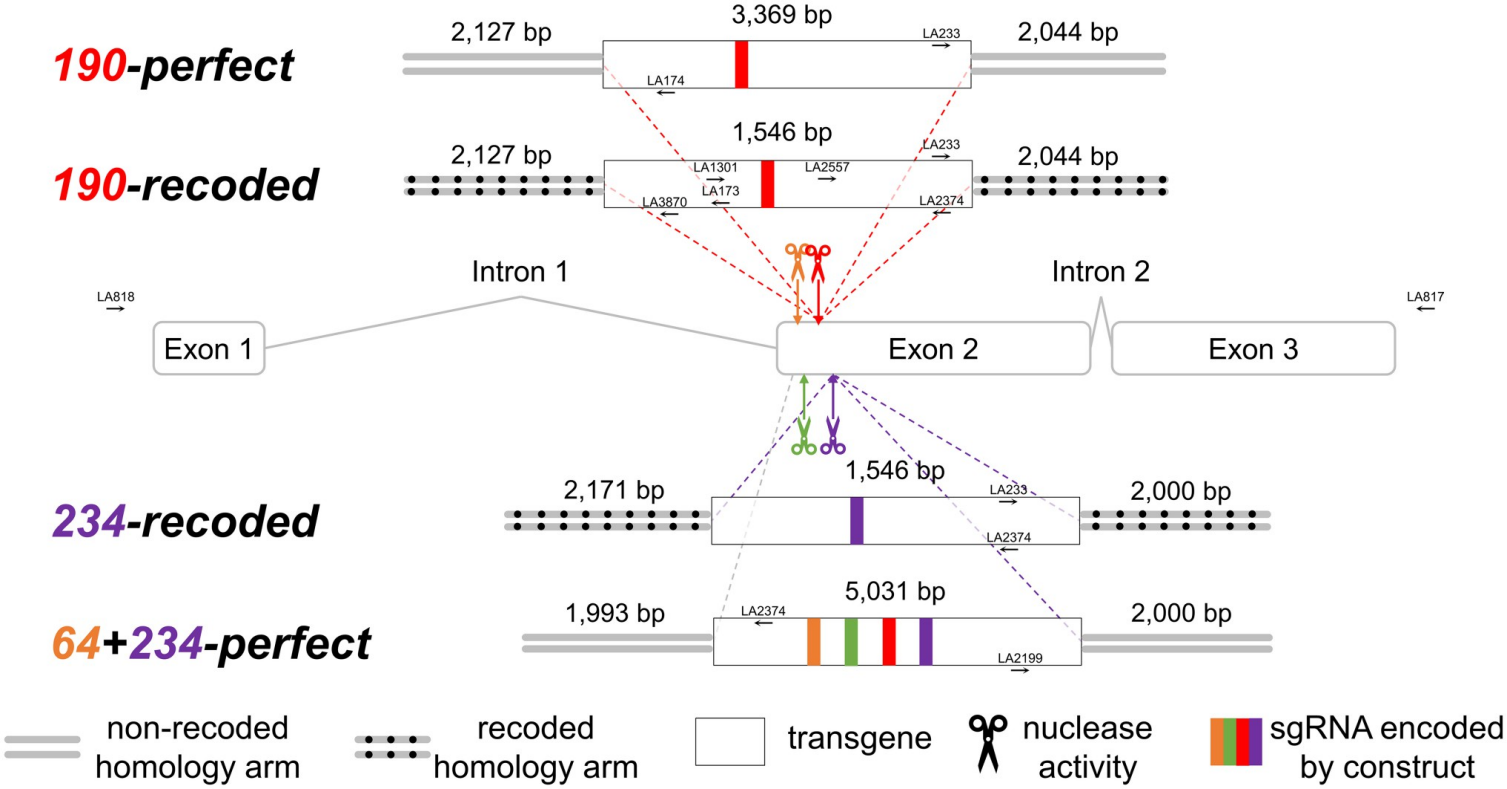
Cut and integration sites on the *Act4* gene. Homology arms of the constructs were designed to match the DSB ends generated by *in vitro* transcribed sgRNAs *64* (orange), *190* (red), and/or *234* (purple). *sgRNA-145* (green), which was not co-injected but encoded in construct *64+234-perfect* is also shown. Activities of all sgRNAs have been validated (Table F in S1 File). Arrows represent primers used to confirm integration site. Figure is not drawn to scale.

To assess the effects on transgene integration of sequence differences between the homology arms of the donor template and recipient chromosome, we compared the integration efficiencies of several plasmid constructs (Fig 1) in *Ae*. *aegypti*. They are named according to the microinjected *in vitro* transcribed sgRNAs: a number based on the position in exon 2 of the DSB they are predicted to generate with Cas9, followed by the type of homology arms (i.e. perfect-match or recoded [intentional substitution of one or more nucleotides of the wild-type sequence]). These constructs were designed to mimic the single (*190-perfect*), classical multiplexing (*64+234-perfect*), and blocking multiplexing (*190-recoded* and *234-recoded*) strategies described for a split-drive system [34]. They comprise homology arms, approximately 2kb each, corresponding to sequence flanking the genomic cut site(s), a fluorescent marker (Hr5/IE1-AmCyan or 3xP3-AmCyan), and sgRNAs compatible to the homology arms for that construct (Fig 1) expressed by an RNA Pol III promoter, but do not encode Cas9. The left and right homology arms contain 1,560 bp and 60 bp of intronic regions, respectively. Both were highly similar (>99.9%) to the LVP reference sequence. Furthermore, the homology arms were sequenced and cloned from our lab strain to reduce the variability between these sequences in the constructs and the target genomic locus. *190-perfect* is comparable to other conventional knock-in constructs in that the homology arms begin immediately at the predicted DSB and that the DNA sequences are identical to the region flanking the DSB. In comparison, the homology arms of *190-recoded* and *234-recoded* also begin immediately at the DSB but nucleotide changes were engineered into them, resulting in a 1.2% sequence heterology (Tables C and D in S1 File) between the homology arm sequences and the DSB flanks. Finally, a 177 bp-long region between positions 57 and 234 of exon 2 was removed from the homology arms of *64+234-perfect* so that the 5’ and 3’ homology arms begin close to the cut sites of the two sgRNAs. Two other sgRNAs, *145* and *190*, were also encoded in addition to *64* and *234* in construct *64+234-perfect*. All four sgRNAs have been shown to exhibit nuclease activity when coupled with Cas9 protein (Table F in S1 File).

**Fig 2:**
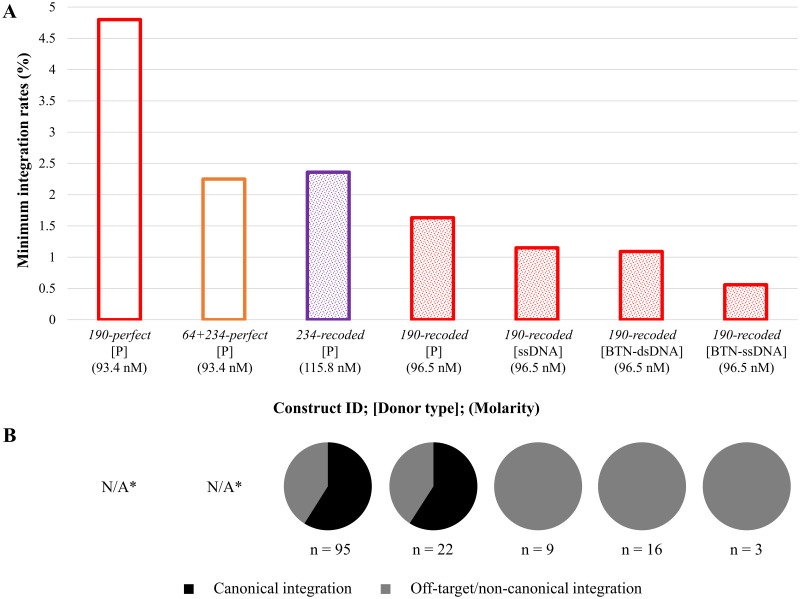
Minimum integration rates and types of integration events as determined by fluorescence and PCR, respectively, for all injected constructs. (A) Bar chart showing minimum integration rates which are calculated as follows: number of positive pools/total number of G_0_ survivors X 100%. Constructs with perfect homology arms are represented by open bars while those with recoded homology arms by filled boxes. P = plasmid. (B) Pie chart showing the proportion of canonical (black) to off-target/non-canonical (grey) integration events for each construct and their corresponding donor types. *N/A = Not applicable. PCR for these constructs were carried out on either pooled samples (n>5) or only one individual G_1_ from each pool (Table E in S1 File) rather than each individual positive G_1_.

Transgenesis events were determined by the detection of fluorescence in G_1_ larvae, using a fluorescence microscope (Fig A in S1 File). The reported minimum integration rates take into account all detectable transgenesis events regardless of whether they were canonical [on target, perfect repair], off-target [different site than intended], or non-canonical [integrated in the target gene but not of the expected structure]. Plasmid construct *190-perfect* was found (Fig 2A and Table A in S1 File) to have the highest minimum integration rate (13/271, 4.80%; 13/13 pools), followed by *64+234-perfect* (8/355, 2.25%; 8/17 pools), *234-recoded* (8/339, 2.36%; 8/16 pools), and *190-recoded* (3/184, 1.63%; 3/9 pools). Using *190-perfect* as the control for comparison, the minimum integration rate was found to be reduced when a mere 1.2% of sequence heterology (Tables C and D in S1 File) was engineered into the homology arms of *190-recoded* (two-sided binomial test, *p* = 0.04, 95% CI) which was co-injected with identical sgRNA. Reductions in integration rates were also observed in *234-recoded* (two-sided binomial test, *p* = 0.03, 95% CI) and *64+234-perfect* (two-sided binomial test, *p* = 0.02, 95% CI) when compared to *190-perfect*, both of which were co-injected with different sgRNAs to *190*. Additionally, a simple binomial probability analysis was carried out taking into account the pooling of G_0_ crosses and observed pool positivity rates to estimate the underlying individual G_0_ integration rates and the statistical significance of the differences observed (S2 and S3 Files). The analysis showed that while there is a substantial overlap of individual G_0_ integration rates that could have resulted in the observed pool positivity rates of *64+234-perfect*, *234-recoded*, and *190-recoded*, no individual G_0_ integration rates would have likely given rise to both 13/13 positive pools and any of the other observed pool positivity rates. The analysis also suggests that the minimum integration rate calculated for *190-perfect* (4.80%) is likely an underestimate of the actual individual G_0_ integration rate.

### Donor types strongly affected the rate of canonical integration

As integration rate was found to be the lowest with plasmid *190-recoded*, several components of the injection mixes were altered to improve the integration rates of construct *190-recoded*. Fusion of Cas9 to a monomeric streptavidin (Cas9-mSA mRNA), when co-injected with a biotinylated dsDNA (BTN-dsDNA) donor, was previously shown consistently to increase HDR efficiency in five different loci in mouse embryos [14]. Since Cas9-mSA protein is not commercially available, we used mRNA as a helper. Helper concentrations are determined in large part by the effect on viscosity of the injection mix at higher concentrations; we used Cas9-mSA mRNA at 365.8 nM or Cas9 protein at 1800 nM. Single-stranded DNA donor templates have also been shown to be efficient in integrating short knock-ins (<25 bp) in both human cells [35] and zebrafish embryos [31]. We, therefore, attempted to optimise HDR-based integration in *Ae*. *aegypti* by testing these methods. Screening of G_1_ larvae for fluorescence indicated that the ssDNA, BTN-dsDNA, and BTN-ssDNA donors of *190-recoded* had generated minimum integrations rates of 1.15% (2/174), 1.09% (2/184), and 0.56% (1/178), respectively (Fig 2A). Note that both ssDNA donors for *190-recoded* have significantly shorter homology arms (617–676 bp each side) than their plasmid and dsDNA counterparts (2044–2126 bp each side) due to limitations in generating long ssDNA.

To verify the integration sites, we conducted PCR on all fluorescent-positive individual G_1_ adults from the recoded constructs and one fluorescent-positive individual/pooled G_1_ sample from each positive pool of the non-recoded constructs (Table E in S1 File). The primer pairs for the PCR were designed such that one would bind to the transgene while the other binds to a genomic region outside of the homology arms. Samples were therefore deemed to have undergone canonical integrations if PCR amplicons of correct sizes were found by gel electrophoresis. Otherwise, they were considered to be either non-canonical or off-target integrations, as the PCR would not be able to differentiate one from the other. For plasmid donors, the proportions of canonical integrations for *190-perfect*, *64+234-perfect*, *234-recoded*, and *190-recoded* were 83%, 88%, 59% and 59%, respectively (Fig 2B). However, the PCR was carried out on every fluorescent-positive adult individually for the recoded constructs and only on a representative fluorescent-positive individual/pooled adults for the *perfect* constructs (Table E in S1 File). Consequently, the proportions between the recoded and non-recoded constructs are not directly comparable due to a potential overestimation of canonical integrations in the latter. In striking contrast to these results, no fluorescent-positive G_1_ individuals generated from ssDNA, BTN-dsDNA, and BTN-ssDNA donor templates of *190-recoded* represented canonical integrations. Both the ssDNA (0/9) and BTN-dsDNA (0/16) *190-recoded* donors were found to have generated significantly lower (two-sided binomial test, *p* < 0.01, 95% CI) number of G_1_ offspring with canonical integrations compared to the plasmid donor (13/22) while no significant difference was detected from the BTN-ssDNA donor (0/3, two-sided binomial test, *p* = 0.07, 95% CI) likely due to the low number of independent G_1_ transgenics generated with this donor. Our PCR assay provides a positive test for a canonical integration but does not rule out the possibility of other integrations, perhaps comprising only part of the donor template, in the same individual. In terms of fluorescent expression patterns, the intensity of fluorescence from transgenic larvae established from the BTN-dsDNA and ssDNA donors appeared distinct from larvae that had the transgene integrated into *Act4* (Fig B in S1 File), a further indication that these represent different molecular events.

### Characterisation of off-target/non-canonical integrations suggest most were outside *Act4* gene

To further characterise the integration sites of individuals shown by PCR to have off-target/non-canonical integrations, a total of 11 individuals were outcrossed to LVP to establish transgenic isolines derived from each G_0_ pool (Table 1). Fluorescent-positive G_2_ mosquitoes were then selected from each isoline for adaptor ligation-mediated PCR, aiming to identify genomic sequence immediately adjacent to the transgene integration site. None of the G_2_ larvae generated from the BTN-ssDNA isoline was observed to express any fluorescence suggesting that the fluorescence observed in the G_1_ founder was likely due to expression from donor template carried over into the embryo by its G_0_ parent. Adaptor ligation-mediated PCR was therefore only performed for the other ten isolines.

**Table 1:** Isolines established from G_1_ individuals and scoring of flight ability of trans-heterozygous females to test for allelism to *AeAct4*^hdr1^.

Construct ID	Donor type	Pool ID	# of isolines	Isoline tested for allelism	Trans-heterozygous females		
					Total scored	Total flightless	% flightless
*234-recoded* *	Plasmid	H	1	H	58	58	100.0
*190-recoded*	BTN-dsDNA	G	3	G1	49	0	0.0
				G2	58	1	1.7
				G3	57	0	0.0
	ssDNA	C	7	C1	50	0	0.0
				C2	52	0	0.0
	BTN-ssDNA	E	1**	-	-	-	-
Total			12	6	-	-	-

*Determined by PCR and sequencing to be canonical integration and used here as a positive control for the allelism test

**Fluorescence was not observed in G_2_ larvae produced from the G_1_ founder individual.

Out of the ten isolines that were analysed, sequence information for seven *190-recoded* (ssDNA) isolines, all generated from a single pool, was obtained. The sequences were all identical, suggesting that these founders originated from a single integration event. We identified a 17 bp genomic sequence (5’-CATGAGTCCTCTCATGG-3’) joined to the 5’ end of the 5’ homology arm of the donor (distal to the transgene), suggesting that the 5’ homology arm of the donor was integrated into the genomic chromosomal locus concurrently with the transgene in the following orientation: genomic locus-CATGAGTCCTCTCATGG-5’ homology arm. A subsequent BLAST query with this 17-bp genomic sequence identified multiple loci in chromosome 1 with 100% sequence similarity in the *Ae*. *aegypti* genome, none adjacent to sequence identical to the homology arm. In all other cases, adaptor ligation-mediated PCR with three different enzymes failed to amplify DNA from the integration site.

Five of these isolines were further tested for allelism with the *Act4* locus by crossing them to *AeAct4*^hdr1^, a previously characterised loss of function integration in *Act4* [24]. As *AeAct4*^hdr1^ larvae express a different fluorescent marker to those generated in this study, trans-heterozygotes could be identified and assessed for the characteristic *AeAct4* mutant phenotype—recessive loss of flight ability in females. For integrations that disrupt the gene, even if not of the canonical structure, trans-heterozygous females should be flightless, as observed previously [24]. As a positive control, an isoline with a canonical integration of *234-recoded* was crossed to the *AeAct4*^hdr1^ line; 100% (58/58) of their trans-heterozygous female progeny were observed to be flightless, as expected. In contrast, only one of 266 (0.4%) trans-heterozygous females generated from the BTN-dsDNA and ssDNA lines was non-flying (Table 2). Since non-flying females were previously recorded at 0.9% in a wild-type population using this assay [36], we conclude that none of these insertions disrupt the *Act4* gene.

**Table 2:** Concentration of each component in injection mixes.

Donor template				Cas9			sgRNA		
Construct	Type	Length (bp/nt)	Molarity (μM)	Type	Length (bp/nt)	Molarity (μM)	sgRNA	Length (nt)	Molarity (μM)
*190-perfect*	Plasmid	10395	0.093	Protein	N/A	1.8	*190*	96	1.3
*190-recoded*	Plasmid	8387	0.096	Protein	N/A	1.8		96	1.3
	BTN-dsDNA	5836	0.096	Cas9-mSA	5103	0.37		96	1.3
	ssDNA	2842	0.096	Protein	N/A	1.8		96	1.3
	BTN-ssDNA	2842	0.096	Cas9-mSA	5103	0.37		96	1.3
*234-recoded*	Plasmid	8387	0.12	Protein	N/A	1.8	*234*	96	1.3
*64+234-perfect*	Plasmid	11923	0.093	Protein	N/A	1.8	*64* + *234*	96 96	1.3 1.3

### Synthesis-dependent strand annealing (SDSA) is the predominant pathway for canonical repair events

**Fig 3:**
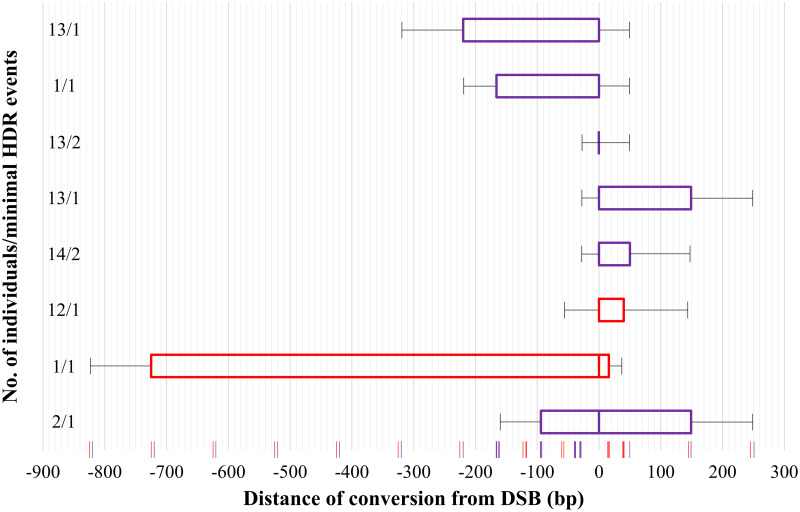
Characteristics of gene conversion tracts in perfect repair events generated with constructs *190-recoded* (red bar) and *234-recoded* (purple bar). Positions of SNPs within the homology arms, relative to the cut sites for *190-recoded* and *234-recoded*, are marked with red and purple vertical lines, respectively (See Tables C and D in S1 File for specific nucleotide changes introduced). Count of HDR events indicates the number of individuals, and independent pools from which a conversion of that size range was recovered. Error bars indicate the range of possible conversion tract lengths which could not be detected by sequencing.

As introduced above, we deliberately included sequence changes in the homology arms of *190-recoded* and *234-recoded* to characterise the length and direction of gene conversion tracts in HDR events in *Ae*. *aegypti*. By sequencing the junction between the cargo of a canonically integrated transgene and its homology arms, we found most integration events to be associated with unidirectional gene conversion (60%), followed by non-conversion (20%) where no nucleotide changes in the homology arms of the donor plasmid were copied into the genome, and bidirectional gene conversion (20%). All conversion tracts ended within 220–319 bp proximal to the DSB, except in three individuals where bidirectional conversions of up to 725 bp (725 bp 5’ of DSB; 16 bp 3’ of DSB, one individual) and 149 bp (94 bp 5’ of DSB; 149 bp 3’ of DSB, two individuals) proximal to the DSB were observed for *190-recoded* and *234-recoded*, respectively (Fig 3). All conversion events were observed to be continuous, such that each mutation between the outermost converted nucleotide and the predicted DSB was present.

**Fig 4:**
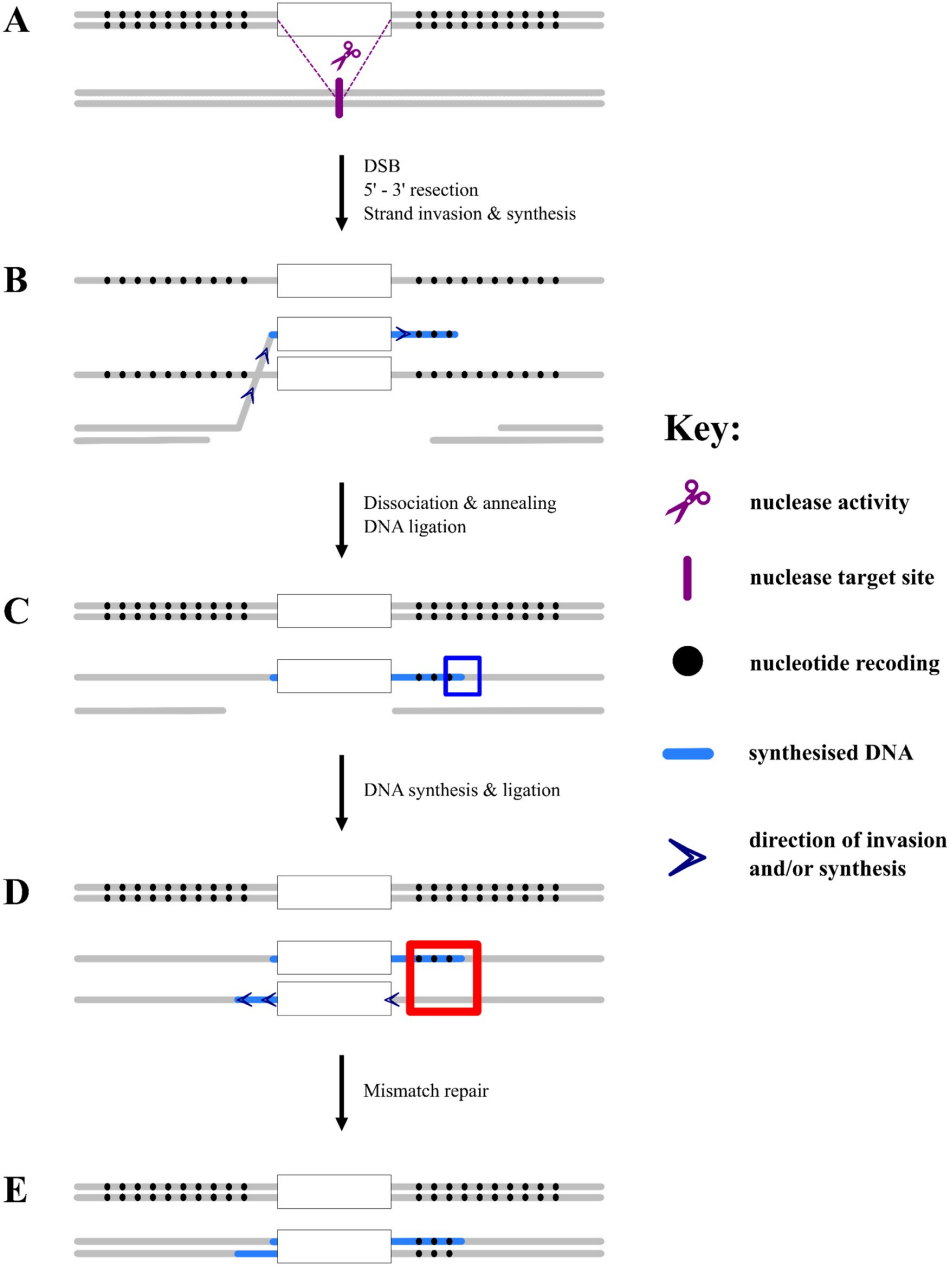
Proposed mechanisms of synthesis-dependent strand annealing (SDSA) and mismatch repair. (A) Double-stranded break caused by Cas9 is followed by 5’–3’ resection of the broken ends, leaving 3’ overhangs which will search for homology to initiate repair. (B) One of the overhanging strands finds homology in the donor and invades very closely to the homology arm-transgene junction, thereby bypassing the recoding on this side of the homology arms. The invading strand synthesises DNA using donor sequences as a template. (C) When this newly synthesised strand recognises homology on the 3’ overhang of the other end of the DSB, it dissociates from the template, anneals to the 3’ overhang, and is ligated to the 5’ end of one of the previously resected strands (squared in blue). (D) The other strand now repairs its break by DNA synthesis and ligation using the invading strand as its template, forming a heteroduplex region (squared in red). (E) Finally, the heteroduplex region is resolved by the mismatch repair pathway which favours the invading strand over the non-invading strand.

Of the 10 HDR events characterised, the unidirectionality of the integration of the recoding for the majority (60%) of the events suggests that the SDSA pathway with a one-ended strand invasion is the most likely mechanism through which these integrations had occurred (Fig 4). The remaining 20% of bidirectional gene conversion could be explained either by Holliday junction formation followed by branch migration (non-crossover) or SDSA initiated by two-ended invasion. Similar observations were previously reported in mammalian cells and *D*. *melanogaster* where SDSA, initiated by both one- and two-ended invasions, was implicated as the pathway for HDR [26,28]. Interestingly, an increase in gene conversion bidirectionality was also associated with longer gene conversion tracts in both studies [27,28] and this process seemed to be regulated by the mismatch repair mechanism in mammalian cells [26].

## Discussion

This study provides insights into various aspects of Cas9-stimulated homology-directed repair in *Ae*. *aegypti*. To control for variability between genomic loci and homology arm length which may affect integration rates, our experiments involved a single target locus–*Act4* and used a limited range of homology arm variants. Further investigations at other target loci and with varying homology arms lengths will therefore be needed for the conclusions in this study to be generalised. The integration rates observed in this study are largely similar to the integration rates (~0.4% to ~3.5%, with an outlier of 30.8%) observed from two other Cas9-directed transgene insertion studies in *Ae*. *aegypti* [37,38]. It is however challenging to infer any factors that may have caused any differences in integration rates between those studies and the present study as the former targeted a total of seven different genomic loci for HDR. For *Anopheles*, integration rates were found to be at a somewhat higher range (2.8% to 12.5%) in the one comparable study ([18], other studies differed significantly in experimental methods, e.g. using only G_0_ showing transent expression, or plasmid-expressed Cas9 rather than Cas9 protein, e.g.[17,39–44]).

In these experiments we wanted to understand HDR efficiency in relation to donor template sequence heterology as generating transgenic strains which require some sequence heterology (i.e. multiplexing strains for HEG drive) may be more onerous than was previously observed. Although we cannot rule out the possibility that other microinjection-associated factors may have caused differences in integration efficiencies, we have tried as much as possible to control them. All the microinjections in this study were performed by the same highly skilled team of four injectors. The high G_0_ survival rates allowed us to have a high number of G_0_ pools, which would lessen any bias caused by small sample sizes. We observed at least a 2-fold reduction in integrations in *190-recoded* (cargo size = 1,546 bp) when compared to *190-perfect* (cargo size = 3,325 bp), which may be due to the 1.2% sequence heterology that was introduced into the homology arms of the former construct (Fig 1). Since our estimate of integration rate would not be able to detect multiple integration events within the same pool, especially for *190-perfect* where all G_1_ pools returned positives, this is likely a significant underestimate of the magnitude of the effect. Our results are in fact consistent with findings from mouse cells and the fruit fly, *D*. *melanogaster*, where sequence heterologies of <1.5% in the homology arms of the donors were found to have caused significant reductions in HDR as compared to donors with homology arms identical to the recipients [26–28]. This suggests that the HDR pathways, at least in mice, *D*. *melanogaster*, and *Ae*. *aegypti*, are highly sensitive to sequence heterology however, it is unknown if the pattern of the mismatches is significant. Our constructs *190-recoded* and *234-recoded* have a ~50 bp-long uninterrupted perfect homology immediately 5’ and 3’ to the cut site, respectively (Tables C and D in S1 File). If this sensitivity to sequence heterology is fundamental to HDR, in addition to the known issue of sequence variation in the sgRNA recognition sequence [45–49], sequence variation in the proximal genomic region may significantly affect homing rates, perhaps representing refractory or homing-resistant sequences in some cases.

The reduction in minimum integration rate observed in *234-recoded* and *64+234-perfect* could potentially be due to a difference in the cutting efficiency between the sgRNAs used for each construct. Various studies and our observations have demonstrated that the cutting efficiencies of different sgRNAs, even when expressed at similar levels, could be affected by their inherent ability to cause DSBs and/or the chromatin structure of the target site [24,50–52]. Alternatively, or additionally for *64+234-perfect*, this decrease could be caused by an additional complication for the DNA repair pathways to identify homology in *64+234-perfect* and recipient chromosomes. A region of extraneous ‘unmatched’ homology will be retained in the cut chromosome whenever *sgRNA-64* and *sgRNA-234* do not produce cuts simultaneously. This could be exacerbated if *sgRNA-145* and *sgRNA-190* which are encoded in the construct were expressed and mediated cuts. Even when simultaneous cuts occur, there will still be a 7-bp extraneous region upstream of the *sgRNA-64* cut site. The effects of such ‘unmatched’ homology were demonstrated to decrease HDR efficiency in *D*. *melanogaster* [53,54]. From the two studies conducted in *D*. *melanogaster*, the distance between two cut sites appears to have affected the occurrence of simultaneous cuts [55,56]. Four sgRNAs designed to target a region of ~2.2kbp were found to predominantly produce deletions (simultaneous cuts) while four other sgRNAs targeting a region of ~250bp made only single cuts, suggesting that the frequency of simultaneous cuts reduces with the distance between cut sites. Here, we have four sgRNAs targeting a region of 170bp. We, therefore, hypothesise that the observed reduction in HDR rate for *64+234-perfect* relative to *190-perfect* is due, at least in part, to the need to resect the cut sequence in the genomic copy before finding homology with the injected template. Overall, this highlights the potential complexity surrounding multiplexing designs for homing-based gene drives.

For plasmid templates the majority of integrations were canonical while no canonical integrations were generated from any of the alternative donor types (Table 2 and Fig 2). The PCR test used identifies integrations at the expected site and orientation, while the test for allelism additionally recognises more complex insertions at the *Act4* locus, e.g. larger indels, which do not necessarily contain the primer binding sites in the expected locations. Based on these tests, most of these events did not generate *Act4* loss-of-function mutants and were presumably inserted elsewhere in the genome. This indicates that the type of repair pathways initiated are affected by donor forms and that the circular double-stranded template is a preferred HDR template in *Ae*. *aegypti*, at least relative to the alternatives that we investigated. Off-target integration events have been shown to occur in mice with microinjection of linear dsDNA, without any nucleases, into pronucleus embryos [57]. Events that integrated only part and not the ends of the donor were found to be dependent on sequence microhomology between the donor and the host genome, while those where at least one end of the donor was integrated could occur with or without microhomology. Microhomology was also implicated in off-target integration of DNA in other separate studies conducted on mice and *Arabidopsis thaliana* [57–59]. Such off-target integration rates may therefore vary considerably between different template sequences.

Multiple studies have investigated the correlation between donor template forms and the precision and efficiency of HDR at a known nuclease cut site but none have been able to fully characterise the various unintended integrations that could have occurred in those studies [30,32,60–63]. Li et al. [32] demonstrated in human cells that linear dsDNA donors were most efficient in causing (off- and on-target) integration events and ssDNA donors performed significantly better in generating on-target HDR events. A follow-up study found that ssDNA donors tended to generate incomplete integration where one end of the template is perfectly copied into the genome but not the other [61]. In mice, both long (>1,000 nt) and short (<200 nt) ssDNA donors were also shown to produce 3–18% and 20–30% illegitimate/non-canonical integrations, respectively, in injected individuals [63]. Generally, linear dsDNA is recognised as having high efficiency for integrations [62] but also a high tendency to contribute to off-target integrations [60]. Off-target integrations were successfully mitigated by modifying (i.e. biotinylation, linking single-stranded ends with C6-polyethylene glycol, and protein capping) the 5’ ends of linear dsDNA templates [30,60,64] but this mitigation was not observed with the biotinylated donors used here. Based on our data, to generate on-target knock-ins in *Ae*. *aegypti*, use of plasmid templates appears superior to the various alternatives tested, preferably using homology arms closely identical to the genomic sequence of the recipient strain.

## Materials and methods

### Mosquito rearing

Liverpool (LVP) strain *Aedes aegypti* [24] were used for outcrossing and as the injection strain. LVP and transgenic lines were maintained at 28°*C*, 70% relative humidity on a 12/12 hour light/dark cycle with 1 hour of dawn and dusk, and provided with 10% sucrose solution *ad libitum*. Larvae were vacuum hatched in water containing Interpet Liquifry no. 1 (Interpet, Surrey, UK) and then reared in pans and fed with ground TetraMin flakes (Tetra, Herrenteich, Germany). Cages of adult mosquitoes were blood fed with defibrinated horse blood (TCS Bioscience) using a Hemotek membrane feeding system (Hemotek), with the reservoir covered with parafilm (Merck). Eggs were collected onto wet coffee filter paper and stored dry until hatched.

### Construct design

All homology arms were designed based on the *Act4* genomic sequence obtained from our laboratory LVP strain. Construct *190-perfect* was assembled with the NEBuilder HiFi DNA Assembly Master Mix (NEB) using four fragments either synthesised (Twist, California, United States) or amplified from existing plasmids (Text A in S1 File). *64+234-perfect* was made by ligating linearised with restriction enzymes *Ngo* MIV and *Sac* II. The homology arms of *190-recoded* and *234-recoded* were recoded (Tables C and D in S1 File) and these constructs were synthesised as plasmids (Genewiz, Massachusetts, United States). DNA sequences of primers, synthesised fragments and plasmids used for designing these constructs are listed in Table B and Text A in S1 File.

### Donor template preparation

Plasmid constructs were prepared with NucleoBond Xtra Midi EF (Macherey-Nagel) according to the manufacturer’s protocol and Sanger sequence confirmed (Eurofins Genomics, Ebersberg, Germany). The antisense (also the strand that *sgRNA-190* binds to) ssDNA (primers: 5’-phosphorylated LA587 and LA588) and BTN-ssDNA (primers: 5’-phosphorylated LA587 and 5’-biotinylated LA588) donors of *190-recoded* were generated using the Guide-it Long ssDNA Production System (Takara Bio). Biotinylated donors were amplified from their respective plasmid backbones with 5’-biotinylated primers LA2967 and LA2968. All non-plasmid donors were gel extracted and purified using the NucleoSpin Gel and PCR Clean-up kit (Macherey-Nagel) and ethanol precipitated before injection mix preparation.

### RNA synthesis

Plasmid pCS2+Cas9-mSA (Addgene plasmid #103882), a kind gift from Janet Rossant [14] was linearized with *Not* I and used as a template for *in vitro* transcription with the mMESSAGE mMACHINE Sp6 Transcription kit (ThermoFisher) according to the manufacturer’s instructions. RNA was purified using the MEGAClear Transcription Clean-Up Kit (ThermoFisher) and further ethanol precipitated. sgRNA templates were prepared as described in Bassett et al. [51], with primers LA137 and LA138 (*sgRNA-64*), LA137 and LA2676 (*sgRNA-145*), LA137 and LA139 (*sgRNA-190*), LA137 and LA140 (*sgRNA-234*). sgRNAs were transcribed using the MEGAScript T7 Transcription Kit (ThermoFisher) and purified using the MEGAClear Transcription Clean-Up Kit (ThermoFisher).

### Embryo microinjections

LVP strain eggs were injected as per [65] with the following modifications. Mosquitoes were allowed to lay eggs in the dark for approximately 35 minutes, before being replaced into the injection cage. Five days post injection, embryos were hatched in a vacuum hatcher to encourage simultaneous hatching. Injection mixes are shown in Table 2.

### Assessing nuclease activity of *sgRNA-145* in the presence of Cas9 protein

Two replicates of ~100 LVP eggs were injected as described above with 300ng/μL of Cas9 protein (PNA Bio) and 100 ng/μL of *in vitro* transcribed *sgRNA-145* and were allowed to develop for approximately 24 hours. Genomic DNA were then extracted using the Nucleospin Tissue Kit (Macherey-Nagel). Amplicon sequencing was carried out on the genomic DNA as previously published [38]. Approximately 300 bp surrounding the sgRNA target site was amplified using primers LA2615 and LA2616 listed in Table B in S1 File. A second round of PCR was performed using the Nextera XT index kit, and Nextera XT index kit D (Illumina). Amplicon sizes were verified on a Tapestation using High Sensitivity D1000 Screen tapes (Agilent). The NEBNext Library Quant kit (NEB) was used to quantify the amplicons prior to pooling. Sequencing was carried out by the Bioinformatics, Sequencing and Proteomics facility at The Pirbright Institute. The Illumina Miseq reads were first checked for sequence quality using FastQC [66]. The low-quality regions and sequencing adapters were trimmed using the Trimmomatic tool [67]. Trimmed reads were then analysed using CRISPResso2 [68] to determine cut rates in the injected eggs.

### Determination of minimum integration rates

Injection survivors, termed generation 0 (G_0_), were reared to adulthood as above. G_0_ females were crossed in groups of ~20 individuals per cage with equal numbers of LVP strain males. Each cage was deemed a separate pool. G_0_ males of up to one week post eclosion were first individually crossed in separate containers with four to five LVP females. This was to allow each individual male to have an opportunity to mate. After at least two days, mosquitoes within approximately 20 of the containers were released into a cage, so that each cage contained approximately 20 males and 80–100 females. Again, each cage was deemed a separate pool. The cages were bloodfed every 4 to 5 days and at least four ovipositions of eggs were collected, which were termed generation 1 (G_1_). G_1_ L4/pupae were screened under a Leica M165C fluorescent microscope. *190-perfect* and *64+234-perfect* pools were screened up to the fourth oviposition or until at least one positive individual was identified while *190-recoded* and *234-recoded* pools were screened to oviposition four to obtain the maximum number of positive individuals. Minimum integration rates for each construct were calculated as follows:

$$\frac{\text{Number}\;\text{of}\;\text{positive}\;\text{pools}}{\text{Number}\;\text{of}\;\text{surviving}\;\text{G}0}\;\text{X}\;100\%$$

This is ‘minimum’ as a pool with multiple positive G_1_ may represent multiple independent insertions—this likely provides a substantial underestimate at higher integration frequencies, e.g. *190-perfect*, where all pools contained positives.

### Confirmation of insertions

Selected fluorescent positive adults generated with the *perfect* constructs were snap-frozen in liquid nitrogen in their pools and/or individually (Table E in S1 File) while all positive adults from the *recoded* constructs were snap-frozen individually. Genomic DNA was extracted from adult mosquitoes identified as positive through screening, using Nucleospin Tissue Kit (Macherey-Nagel). PCR reactions were carried out with primer pairs LA818 and LA174/LA2374, LA2199/LA2374 and LA817 (Fig 1), which spanned across the insertion and the expected genomic region outside of the insert homology arm sequences. PCR products were separated and visualised on 1% agarose (Merck)/TAE (ThermoFisher) gel to confirm insertions were in the expected genomic region. Among samples that did not show the expected amplicon size from both PCR reactions, a total of 11 G_1_ males positive for *190-recoded* from four pools were selected and crossed individually to five LVP females. Adaptor ligation-mediated PCR was performed according to previously published method [69] on G_2_ progeny from these crosses, with several modifications, in order to determine the genomic integration sites through amplification of the flanking sequences. Genomic DNA was digested with *Nco* I, *Bsp* HI and *Pci* I restriction enzymes and each digested DNA was ligated to an *Nco* I adaptor (prepared by annealing LA179 and LA1703) using T4 DNA ligase (NEB). Following primary (LA187 and LA173/LA1301) and semi-nested (LA187 and LA3870/LA2557) PCRs, the products were separated on 1% agarose/TAE gel and selected bands were excised. Amplicons were purified using Nucleospin Gel and PCR cleanup kit (Macherey-Nagel) and Sanger sequenced (Eurofins) with primers LA989/LA233. For pools in which the insertion locus could not be identified by adaptor ligation-mediated PCR, adults were crossed to line *AeAct4*^hdr1^ [24] and blood fed. Eggs were collected and vacuum hatched. Pupae were screened under a Leica M165C fluorescent microscope for the presence of both *190-recoded*/*234-recoded* and *AeAct4*^hdr1^ markers. Female trans-heterozygotes were retained, and flight ability was assessed by observation two to four days post eclosion. Flight was encouraged during observation through tapping of the area where the mosquito rested, as per previous assessments with *AeAct4*^hdr1^ [22]. Non-flying adults were reassessed one to two days after the first assessment to confirm their flight status. Adults which displayed no flight on both assessments were deemed non-flying.

The views, opinions and/or findings expressed are those of the authors and should not be interpreted as representing the official views or policies of the U.S. Government.

## Supporting information

S1 File Supplementary information. Table A. Integration rates of various constructs and respective donor templates. Table B. List of primer sequences used. Table C. Positions of SNPs introduced in the homology arms of 190-recoded. Table D. Positions of SNPs introduced in the homology arms of 234-recoded. Table E. PCR results to confirm integration from constructs 190-perfect and 64+234-perfect. Table F. Nuclease activity of sgRNAs. Text A. DNA sequences of plasmids/synthesised fragments in GenBank/fasta format. Fig A. Fluorescence patterns of hr5/ie1-AmCyan (64+234-perfect and 190-perfect) and the eye-specific 3xP3-AmCyan (190-recoded and 234-recoded) with canonical integrations (as determined by PCR) into the Act4 locus. Fig B. Different fluorescence intensity of 3xP3-AmCyan expression observed in different isolines. Isoline H is a canonical integration generated with plasmid 234-recoded while the isolines C1.2 and G2 are off-target integrations generated with ssDNA and BTN-dsDNA donors of 190-recoded. Isoline G2 gives consistently stronger expression of the 3xP3-AmCyan marker than H, while C1.2 is weaker.
(DOCX)

S2 File Statistical analysis of pool positivity. (DOCX)

S3 File R script for statistical analysis. (TXT)
