# Supplementary material for: Considerations for homology-based DNA repair in mosquitoes: Impact of sequence heterology and donor template source
Source: PLoS Genet. 2022 Feb 18;18(2):e1010060. doi: 10.1371/journal.pgen.1010060 (PMC8893643; doi:10.1371/journal.pgen.1010060)
Supplement: S1 File — Table A. Integration rates of various constructs and respective donor templates. Table B. List of primer sequences used. Table C. Positions of SNPs introduced in the homology arms of 190-recoded. Table D. Positions of SNPs introduced in the homology arms of 234-recoded. Table E. PCR results to confirm integration from constructs 190-perfect and 64+234-perfect. Table F. Nuclease activity of sgRNAs. Text A. DNA sequences of plasmids/synthesised fragments in GenBank/fasta format. Fig A. Fluorescence patterns of hr5/ie1-AmCyan (64+234-perfect and 190-perfect) and the eye-specific 3xP3-AmCyan (190-recoded and 234-recoded) with canonical integrations (as determined by PCR) into the Act4 locus. Fig B. Different fluorescence intensity of 3xP3-AmCyan expression observed in different isolines. Isoline H is a canonical integration generated with plasmid 234-recoded while the isolines C1.2 and G2 are off-target integrations generated with ssDNA and BTN-dsDNA donors of 190-recoded. Isoline G2 gives consistently stronger expression of the 3xP3-AmCyan marker than H, while C1.2 is weaker. (DOCX) [file pgen.1010060.s001.docx]

Table A. Integration rates of various constructs and respective donor templates.

| **Construct ID** | **Donor**  **template** | **Number of** | | | | **Total**  **screened** | **Minimum integration**  **rate**  **(%)** |
| --- | --- | --- | --- | --- | --- | --- | --- |
|  |  | **G_0_** | **Pool** | **Positive^a^**  **pool** | **Positive^b^**  **G_1_** |  |  |
| *190-perfect* | Plasmid | 271 | 13 | 13 | 350 | 9774 | 4.80 |
| *64+234-perfect* | Plasmid | 355 | 17 | 8 | 207 | 22158 | 2.25 |
| *234-recoded* | Plasmid | 339 | 16 | 8 | 140 | 26891 | 2.36 |
| *190-recoded* | Plasmid | 184 | 9 | 3 | 24 | 17525 | 1.63 |
|  | BTN-dsDNA | 184 | 10 | 2 | 19 | 20658 | 1.09 |
|  | ssDNA | 174 | 9 | 2 | 10 | 10282 | 1.15 |
|  | BTN-ssDNA | 178 | 10 | 1 | 4 | 15014 | 0.56 |

^a^a pool is considered positive if at least one AmCyan-fluorescing individual G_1_ was recovered from the pool

^b^an individual G_1_ is considered positive if it expresses AmCyan fluorescence regardless of integration type

Table B. List of primer sequences used.

| Primer name | Sequence (5’-3’) |
| --- | --- |
| LA137 | AAAAGCACCGACTCGGTGCCACTTTTTCAAGTTGATAACGGACTAGCCTTATTTTAACTTGCTATTTCTAGCTCTAAAAC |
| LA138 | GAAATTAATACGACTCACTATAGGGGAGCACTAGTCATTGACAAGTTTTAGAGCTAGAAA |
| LA139 | GAAATTAATACGACTCACTATAGGGGTCAAAAAGATGCCTACGTGTTTTAGAGCTAGAAA |
| LA140 | GAAATTAATACGACTCACTATAGGGTGCTCTATGGGATATTTCAGTTTTAGAGCTAGAAA |
| LA173 | CAGCGGTTGCCGTACATGAACAC |
| LA174 | AGGATGTCGAAGGAGAAGGCCAGG |
| LA179 | GTGTAGCGTGAAGACGACAGAAAGGGCGTGGTGCGGAGGGCGGTG |
| LA233 | ACTGGGGTAACCTTTGAGTTCTC |
| LA587 | AACGAGCCCTGTACCTATTGAT |
| LA588 | GTAGACCGTTTCATGAATGCCA |
| LA817 | ACGACCGTCACCCTATTATTTCTC |
| LA989 | GCGCCTCTATTTATACTCCGGC |
| LA818 | GAAACCTCACCCGAACGCAC |
| LA1301 | TGGCCTTCTCCTTCGACATCCTGT |
| LA1703 | CATGCACCGCCCTCCG |
| LA2199 | CGTTCTCTCGAACCACGCTC |
| LA2374 | CTGAGAGAACTCAAAGGTTACCCCAG |
| LA2557 | TTCGAGAAGATGACCGTGTG |
| LA2615 | TCGTCGGCAGCGTCAGATGTGTATAAGAGACAGCCTTGAAGCGGCGCCAAAAT |
| LA2616 | GTCTCGTGGGCTCGGAGATGTGTATAAGAGACAGGTTGGTGATGATACCGTGCTCT |
| LA2676 | GAAATTAATACGACTCACTATAGGTCACACCCTGGTGGCGAGGGGTTTTAGAGCTAGAAA |
| LA2832 | CTAGTGCTCCAGCATCATC |
| LA2833 | TGTGACGATGATGCTGGAGCACTAGTCATTGACAACGGATCCG |
| LA2834 | CTACTCGTAAAGCTAGGCATCTTTTTGACCC |
| LA2835 | AAAAAGATGCCTAGCTTTACGAGTAGAATTCTACGCGTAAAACAC |
| LA2886 | TTGGGGCACTACTCCCAAACGCGCCAGTGGT |
| LA2887 | TGGCGCGTTTGGGAGTAGTGCCCCAACTGGG |
| LA2888 | ATGATACCGTGCTCTATGGGATATTTCAGGGTGAGGATACCTC |
| LA2889 | AATATCCCATAGAGCACG |
| LA2967 | CGCCAGGGTTTTCCCAGTCACGAC |
| LA2968 | TCACACAGGAAACAGCTATGAC |
| LA3870 | CCCTTCACGGTGAAGTAGTG |

Table C. Positions of SNPs introduced in the homology arms of *190-recoded*.

| Sequence | Distance of recoding from cut site (bp) | | | | |  | Distance of recoding from cut site (bp) | | |
| --- | --- | --- | --- | --- | --- | --- | --- | --- | --- |
|  | 1 SNP approximately every 100 bp upstream  of the nearest SNP. | 122 | 118-117 | 50 | 48 |  | 14-16 | 38-40 | 1 SNP approximately every 100 bp downstream  of the nearest SNP. |
| LVP |  | G | CC | C | C |  | GCC | ACC |  |
| *190-recoded* |  | T | GA | T | A |  | TGA | TGA |  |

Position of cut site (0 bp) is highlighted in red.

Table D. Positions of SNPs introduced in the homology arms of *234-recoded*.

| Sequence | Distance of recoding from cut site (bp) | | | | | | |  | Distance of recoding from cut site (bp) | |
| --- | --- | --- | --- | --- | --- | --- | --- | --- | --- | --- |
|  | 1 SNP approximately every 100 bp upstream  of the nearest SNP. | 220 | 166-165 | 162-161 | 94-92 | 40-38 | 31-29 |  | 50 | 1 SNP approximately every 100 bp downstream  of the nearest SNP. |
| LVP |  | T | GG | CC | CGC | GGT | GCC |  | G |  |
| *234-recoded* |  | A | TA | AA | TAA | TAA | TAA |  | A |  |

Position of cut site (0 bp) is highlighted in red.

Table E. PCR results to confirm integration from constructs *190-perfect* and *64+234-perfect*.

| Construct | Pool | Genomic DNA source | PCR result |
| --- | --- | --- | --- |
| *190-perfect* | A | 1 adult | + |
|  | B | 1 adult | + |
|  | C | 1 adult | + |
|  | D | 1 adult | + |
|  | E | Not done* | Not done |
|  | F | 1 adult | - |
|  | G | 1 adult | + |
|  | H | 1 adult | + |
|  | I | 1 adult | + |
|  | K | 1 adult | + |
|  | P | Pool of female adults | + |
|  | R | Pool of female adults | - |
|  | U | 1 adult | + |
| *64+234-perfect* | A | Pooled adults | - |
|  | E | Pooled adults | + |
|  | G | 1 adult | + |
|  | P | 1 adult | + |
|  | S | 1 adult | + |
|  | T | 1 adult | + |
|  | W | Pooled adults | + |
|  | Y | 2 male adults | + |

*fluorescent-positive G_1_ mosquitoes from this pool were not collected for DNA extraction.

Table F. Nuclease activity of sgRNAs.

| sgRNA | Method | Replicate | Nuclease activity  (Yes/No) | Rank within  species | Cut rate  (%) | Source  of result |
| --- | --- | --- | --- | --- | --- | --- |
| *64* | *in vitro* Cas9 cleavage | 1 | Yes | 2^nd^ | N/A | [1] |
| *190* |  | 1 | Yes | 1^st^ | N/A |  |
| *234* |  | 1 | Yes | 1^st^ | N/A |  |
| *145* | Illumina Miseq and  analysis with CRISPResso2 | 1 | Yes | N/A | 34.2 | Present  study |
|  |  | 2 | Yes | N/A | 8.8 |  |

N/A = not applicable

Text A. DNA sequences of plasmids/synthesised fragments in GenBank/fasta format.

LOCUS AGG1235 12602 bp ds-DNA circular 26-JUL-2021

DEFINITION .

FEATURES Location/Qualifiers

CDS complement(33..893)

/label="AmpR - BsaI Recoded"

misc_feature complement(894..998)

/label="AmpR Promoter"

CDS complement(1101..1418)

/label="Eco47I/T7 (CUT) (2)"

misc_feature 1419..3411

/label="5'HomArm - AeAct4 (AAEL001951) (7bp SHORT)"

misc_feature 1458..1795

/label="Exon 1 - AeAct4 (AAEL001951)"

misc_feature 1796..3354

/label="Intron 1 - AeAct4 (AAEL001951)"

5'UTR 3355..3383

/label="5'UTR (2) - AeAct4 (AAEL001951)"

misc_feature 3415..3464

/label="attP50"

misc_feature 3474..4675

/label="Hr5IE1"

misc_feature 4720..4789

/label="adh Intron"

CDS 4825..4851

/label="NLS"

misc_feature 4864..5564

/label="AmCyan"

CDS 5576..5614

/label="NLS (2)"

3'UTR 5633..6414

/label="K10"

misc_feature 6423..7013

/label="U6-1"

misc_feature 6949..6961

/label="Proximal Sequence Element - U61"

misc_feature 7014..7033

/label="Act4-3 (Aegyp)"

misc_feature 7034..7119

/label="gRNA_Backbone-23\with\5nt\extended\upper\stem"

misc_feature 7127..7669

/label="U6-2"

misc_feature 7605..7617

/label="Proximal Sequence Element - U62"

misc_feature 7670..7689

/label="Act4-1 (Aegyp)"

misc_feature 7690..7771

/label="gRNA_Backbone_29"

misc_feature 7779..8313

/label="U6-3"

misc_feature 8250..8262

/label="Proximal Sequence Element - U6-3/7SK"

misc_feature 8314..8333

/label="Act4-2 (Aegyp)"

misc_feature 8334..8413

/label="gRNA_Backbone_09"

misc_feature 8421..8990

/label="Putative 7SK Promoter"

misc_feature 8926..8938

/label="Proximal Sequence Element - U6-3/7SK"

misc_feature 8991..9010

/label="Act4-4 (Aegyp)"

misc_feature 9011..9090

/label="gRNA_Backbone_25"

misc_feature 9122..11121

/label="3'HomArm - AeAct4 (Seq.Corrected)"

misc_feature 9842..9901

/label="Intron 2 - AeAct4 (AAEL001951)"

misc_feature 9902..10762

/label="Exon 3 - AeAct4 (AAEL001951)"

CDS complement(11122..11466)

/label="Eco47I/T7 (CUT) (1)"

misc_feature complement(11495..11511)

/label="Lac Operator"

misc_feature complement(11519..11549)

/label="Lac UV5"

misc_feature complement(11876..12464)

/label="pJet Ori"

ORIGIN

1 taaagtatat atgagtaaac ttggtctgac agttaccaat gcttaatcag tgaggcacct

61 atctcagcga tctgtctatt tcgttcatcc atagttgcct gactccccgt cgtgtagata

121 actacgatac gggagggctt accatctggc cccagtgctg caatgatacc gcgcgaccca

181 cgctcaccgg ctccagattt atcagcaata aaccagccag ccggaagggc cgagcgcaga

241 agtggtcctg caactttatc cgcctccatc cagtctatta attgttgccg ggaagctaga

301 gtaagtagtt cgccagttaa tagtttgcgc aacgttgttg ccattgctac aggcatcgtg

361 gtgtcacgct cgtcgtttgg tatggcttca ttcagctccg gttcccaacg atcaaggcga

421 gttacatgat cccccatgtt gtgcaaaaaa gcggttagct ccttcggtcc tccgatcgtt

481 gtcagaagta agttggccgc agtgttatca ctcatggtta tggcagcact gcataattct

541 cttactgtca tgccatccgt aagatgcttt tctgtgactg gtgagtactc aaccaagtca

601 ttctgagaat agtgtatgcg gcgaccgagt tgctcttgcc cggcgtcaat acgggataat

661 accgcgccac atagcagaac tttaaaagtg ctcatcattg gaaaacgttc ttcggggcga

721 aaactctcaa ggatcttacc gctgttgaga tccagttcga tgtaacccac tcgtgcaccc

781 aactgatctt cagcatcttt tactttcacc agcgtttctg ggtgagcaaa aacaggaagg

841 caaaatgccg caaaaaaggg aataagggcg acacggaaat gttgaatact catactcttc

901 ctttttcaat attattgaag catttatcag ggttattgtc tcatgagcgg atacatattt

961 gaatgtattt agaaaaataa acaaataggg gttccgcgca catttccccg aaaagtgcca

1021 cctgacgtct aagaaaccat tattatcatg acattaacct ataaaaatag gcgtatcacg

1081 aggccgcccc tgcagccgaa ttatattatt tttgccaaat aatttttaac aaaagctctg

1141 aagtcttctt catttaaatt cttagatgat acttcatctg gaaaattgtc ccaattagta

1201 gcatcacgct gtgagtaagt tctaaaccat ttttttattg ttgtattatc tctaatctta

1261 ctactcgatg agttttcggt attatctcta tttttaactt ggagcaggtt ccattcattg

1321 tttttttcat catagtgaat aaaatcaact gctttaacac ttgtgcctga acaccatatc

1381 catccggcgt aatacgactc actataggga gagcggccgc acttccgagt ataaaacccc

1441 ggtaaaccca aggaatcact cacaatcgga ttttgacgct cgctctggta cagttcgata

1501 cggtctagtg aaaccgagga taacgacgaa ggtttttccc cattgatcca ggtcggtgtt

1561 tatgattggt ggaaaaagag ctcgagaaaa gttccatcga agccgttgga aatgtgccgt

1621 cttcctgtga cgtcttgtgg atccagttcc ttgttcacgt ctggtgatcg tgtaaaatgt

1681 gctgtcttgt ggcgtcatat gtgttccaga tccagtgatt acgatccgat gtgatgttga

1741 tcccttgtga acgtcttatc ctgttccgtg tgcaccatgc ataatgtcgt attacgtaag

1801 ttctgaagtg aaacagaaga gtgaattgaa agttttttta ttcaacatca acctaaatat

1861 ggactttact ttccaagaaa attatgcctg atcaactgtg gatagttaca aaaaaaaaag

1921 gtttattaat taaattttat gattacataa tgtgttgaaa agaacaactg aaattttaga

1981 agaagatctt ttcgtgcatc aggctttgcc aattaattga tgataaatta tcatagcaaa

2041 ttaacgtaga gactaaaagg tatatcgtca aatagggctt cttttgacac tattttggca

2101 ttcttgctct ttgagaactt gcaaccctaa aatgggatct tcatcagcct agtggttaga

2161 ttcagcagct acaaagcaaa accatgctga agggttcgat tcccggtcgt ttcaggatct

2221 tttcgtaatt gaaatatcct tgactaccct aagtatcatt gtgcttgcca tttacgaata

2281 tacatattac gatatacgaa tgagaaaatg acaactttgg aaaataaagc tctcaatgtt

2341 tcaataagaa ataaatacta catcagtatt gaaggctaat aacaattaca gattagaacc

2401 tttaaacatc atttctgcaa caggctggat aaagtacagt tggaggatta aattatgcga

2461 ttttgcaatt ttttccgatt aaattcatat ttattcctgg tttggttttt acaaaaaata

2521 tttttacatg acgtttgacc ccgattccct caactttgat tgttatattt ttttttggac

2581 aggttgagtt tgtgggtttt ttcctagtgt tgctttgctt tatgggctct ggttatttaa

2641 aattaaaatt tgacaatctt actacacact ccgaaaaaat catgcgattt tacgtctttt

2701 ggatgcacat aaaagaagcg agccaaatga ggtgaatttg tgtcacattt taaatacgat

2761 ggtgtctgat tcgggaaatg tcaatgatag tgtcattcaa tcataatgtg aattacgtcc

2821 gcagtaattt tcattatttt taagagtgta ctactattta cactacaaaa attttgatac

2881 cccagggggg aacgaggtcc cggatgtcca gctggccaga ttgttggcaa cgagccctgt

2941 acctattgat cgagtcacca aagcactcct caagtgtttt aatctcgacc agacggtgga

3001 cctcggttgt tctcattctc ggagggcgat ttcgcaatca ttagtaccaa ccacatgtcg

3061 aagtcgggag atgttataaa attataacca attattcaaa aaatgacatc attcaatttg

3121 aacaaacgtt cgatagaaat tatatatgat ttcacatgat attaaactac gaagaaaatt

3181 ttacataagg aagtggtata aaacgtaata tgcttaataa aaactttaac ccttttggga

3241 ggataatatt cagaagttct gattcagaac catctctcat gttatgttcg ttttttgttg

3301 cttgtccttt atatgccaca tgaacaataa caccaatatc tatcccattt ccaggaccta

3361 acggaccttg aagcggcgcc aaaatgtgtg acgatgatgc tggagcaCTA Gccaagtagt

3421 gccccaactg gggtaacctt tgagttctct cagttggggg cgtaggtcga caagctttac

3481 gagtagaatt ctacgcgtaa aacacaatca agtatgagtc ataatctgat gtcatgtttt

3541 gtacacggct cataaccgaa ctggctttac gagtagaatt ctacttgtaa tgcacgatca

3601 gtggatgatg tcatttgttt ttcaaatcga gatgatgtca tgttttgcac acggctcata

3661 aactcgcttt acgagtagaa ttctacgtgt aacgcacgat cgattgatga gtcatttgtt

3721 ttgcaatatg atatcataca atatgactca tttgtttttc aaaaccgaac ttgatttacg

3781 ggtagaattc tacttgtaaa gcacaatcaa aaagatgatg tcatttgttt ttcaaaactg

3841 aactcgcttt acgagtagaa ttctacgtgt aaaacacaat caagaaatga tgtcatttgt

3901 tataaaaata aaagctgatg tcatgttttg cacatggctc ataactaaac tcgctttacg

3961 ggtagaattc tacgcgtaaa acatgattga taattaaata attcatttgc aagctatacg

4021 ttaaatcaaa cggacgctcg aggttgcaca acactattat cgatttgcag ttcgggacat

4081 aaatgtttaa atatatcgat gtctttgtga tgcgcgcgac atttttgtag gttattgata

4141 aaatgaacgg atacgttgcc cgacattatc attaaatcct tggcgtagaa tttgtcgggt

4201 ccattgtccg tgtgcgctag tagcatgccc gtaacggacc tcgtactttt ggcttcaaag

4261 gttttgcgca cagacaaaat gtgccacact tgcagctctg catgtgtgcg cgttaccaca

4321 aatcccaacg gcgcagtgta cttgttgtat gcaaataaat ctcgataaag gcgcggcgcg

4381 cgaatgcagc tgatcacgta cgctcctcgt gttccgttca aggacggtgt tatcgacctc

4441 agattaatgt ttatcggccg actgttttcg tatccgctca ccaaacgcgt ttttgcatta

4501 acattgtatg tcggcggatg ttctatatct aatttgaata aataaacgat aaccgcgttg

4561 gttttagagg gcataataaa agaaatattg ttatcgtgtt cgccattagg gcagtataaa

4621 ttgacgttca tgttggatat tgtttcagtt gcaagttgac actggcggcg acaagcaatt

4681 ggtacccggg taggatccta gtgaattcct aatctggcgg taagttgatc aaaggaaacg

4741 caaagttttc aagaaaaaac aaaactaatt tgatttataa cacctttaga aagcgaagtt

4801 gagattcagg ccaccatggg agatcccacc ccacccaaga agaagcgcaa accggctagc

4861 gttatggccc tgtccaacaa gttcatcggc gacgacatga agatgaccta ccacatggac

4921 ggctgcgtga acggccacta cttcaccgtg aagggcgagg gcagcggcaa gccctacgag

4981 ggcacccaga cctccacctt caaagtcaca atggccaacg gcggccccct ggccttctcc

5041 ttcgacatcc tgtccaccgt gttcatgtac ggcaaccgct gcttcaccgc ctaccccacc

5101 agcatgcccg actacttcaa gcaggccttc cccgacggca tgtcctacga gagaaccttc

5161 acctacgagg acggcggcgt ggccaccgcc agctgggaga tcagcctgaa gggcaactgc

5221 ttcgagcaca agtccacctt ccacggcgtg aacttccccg ccgacggccc cgtgatggcc

5281 aagaagacca ccggctggga cccctccttc gagaagatga ccgtgtgcga cggcatcttg

5341 aagggcgacg tgaccgcctt cctgatgctg caaggcggcg gcaactacag atgccagttc

5401 cacacctcct acaagaccaa gaagcccgtg accatgcccc ccaaccacgt ggtggagcac

5461 cgcatcgcca gaaccgacct ggacaagggc ggcaacagcg tgcagctgac cgagcacgcc

5521 gtggcccaca tcacctccgt ggtgcccttc tccggactcc gctcccagat ctcccgaccc

5581 aagaaaaagc ggaaggtgga ggacccgtaa gatccaccgg atctagataa ctggagcttg

5641 ataacattat acctaaaccc atggtcaaga gtaaacattt ctgcctttga agttgagaac

5701 acaattaagc atcccctggt taaacctgac attcatactt gttaatagcg ccataaacat

5761 agcaccaatt tcgaagaaat cagttaaaag caattagcaa ttagcaatta gcaataactc

5821 tgctgacttc aaaacgagaa gagttgcaag tatttgtaag gcacagttta tagaccaccg

5881 acggctcatt agggctcgtc atgtaactaa gcgcggtgaa acccaattga acatatagtg

5941 gaattattat tatcaatggg gaagatttaa ccctcaggta gcaaagtaat ttaattgcaa

6001 atagagagtc ctaagactaa ataatatatt taaaaatctg gccctttgac cttgcttgtc

6061 aggtgcattt gggttcaatc gtaagttgct tctatataaa cactttcccc atccccgcaa

6121 taatgaagaa taccgcagaa taaagagaga tttgcaacaa aaaataaagg cattgcgaaa

6181 actttttatg ggggatcatt acactcgggc ctacggttac aattcccagc cacttaagcg

6241 acaagtttgg ccaacaatcc atctaatagc taatagcgca atcactggta atcgcaagag

6301 tatataggca atagaaccca tggatttgac caaaggtaac cgagacaatg gagaagcaag

6361 aggatttcaa actgaacacc cacagtactg tgtactacca ctggcgcgtt tgggTACGGT

6421 GAggccggcc catgtttcca gactttccct cccggtaaac ggagacaaaa cgacagacgt

6481 aagtaggtac atatgcatac cgcacggaca aatcaaattt gtctggcagc tccaattaga

6541 gtcgttaaaa atttaacgat gcgctaaata acttcaagct atttgtctcg ctggattggc

6601 ttcgagtggt aagatcctat caaatgccga aaacaaaaaa ctttcttctt aattgttcgt

6661 tcttcaacac ctctccatgg tgataacgga tacggtttca ttgtcagcat ccatcctccg

6721 aaaaatacat tacgccttga aatatgcaat cgcaaacacg gatctgtttg gaacatttat

6781 tttactatga agagatgcga taggtaatat ttatttgagc gtttaagata ctcattgttc

6841 tctcaaagaa tgtcattgaa agccaacgag gtcaaatcaa atattataat aaaaaggtca

6901 aagaggacta acttaaagct ctctttatgg ataggaaaaa atattttcgc ccatcgctag

6961 aacttttacc gtttccattg agtatataac taagatgaat gaggctaatt gatgtgctct

7021 atgggatatt tcagttccag agtcgtgctg ggaacagcac gacaagttgg aataaggcaa

7081 gtccgttatc atgccggaag gcaggcaccg attcggtgct ttttttggaa cccgaattta

7141 gtgctatata atttaattcc actagagttt gtatcctttg atagatacgc gtatttcgac

7201 ctcaactgca aggccgtcgt gtactagact tgactaatcc agactggtct tttagttatg

7261 acttctgtcc acatctccat acattcaacg cactgtgcgg ctgtgctgtg cgactccgtc

7321 gagtcgacca acatagttga aacaaattga atatttaatt gatcgttata ggaatggtgt

7381 tagatgagtc atcctttaca gtaagcacat acagtattat aattgaagat cgtcggcaga

7441 taggtgtgta gggtagagta tcagcaataa gttgggacgt ttgacttttt gtaggtagac

7501 aaaaactaaa ctttttttcg cttctctatg tgtgcccccc cgggtagcgt atcgttccga

7561 ttgtggtgcg aacgaatgaa atcgcccatc gagttgatac gtccatccat cgctagaacc

7621 gcgttcgctg tagaagacta tataagagca gaggcaagag tagtgaaatg gagcactagt

7681 cattgacaag tcgcagagca tctgaaaaga tgcaagttgc gataaggcaa gtccgttatc

7741 aagctcggga gagctggcac cgagtcggtg ctttttttga gtctggtaac cctagtgcac

7801 gcaaatatct cgcgggcata tttggttgct gaggtatatt tatatttgaa cgccatgaga

7861 aaaagcggaa gaaattggct catggccgat tttaaggata tttaaaaatt gtacaatgta

7921 catataatag gccaggagaa gtggatgaac ttgtcattca tttttctgtc aattctcata

7981 caaaatctac tttttctttg acataaattc actctaggtg aaccacttcc cctggcctat

8041 taaacatccg ttccttcaat gtgttctttt ttttaagcgt gtgttaaaag tttgctctgc

8101 tggtgaattc acgctctacc cgttcaggca gcattcatcg aaaagcccta tctgctcgca

8161 cacatttaca aaatgctgat tgcgttgtgt gctgaatggg tcactcgtcc gtcactgctt

8221 gctgtgtaca ctgtacagtt acgcagtctg tgcatcgcta gaatcatatt tacggaagag

8281 tattatatat acccgatgcg ttgctcttcg attggtcaaa aagatgccta cgtgtcctag

8341 agccatgaaa atggcaagtt aggataaggc tagtccgtat tcaacgctga aaagcgtggc

8401 accgagtcgg tgcttttttt atgctccatt ctccgccact tgttgatgcg gaccctaacc

8461 acgtggtcgc tcctctgctc accggagcac gtttcataca gcctgacgac gacgagcaat

8521 cagaggtatg gtgagcatgc gcatggagag tggacagcag tgcaccctaa aatcaattca

8581 cacatcatgt gtcaatagct gtgtcaatgt tgcacagcct tttcttatta aatttactcc

8641 ttttgtgacc atttctcttt catccaccgt tattttaatg agttttgtgt tccggtggac

8701 gaacgttcac acaaaaaatg tgtaaatctt aatcaaccag aacacaaagt atagtgaaaa

8761 aattaaagtg tgtggctttt atacatccta actgtaaatt atttttagag tgcgtgcgat

8821 cgttctctcg aaccacgctc tccgctacac attcgcagcg aatggcgtga atggatgaaa

8881 gaacaaacta aagtttattt ttagattcgt ctcaaaacaa ctgctgtgca tcgctagaac

8941 caagaaatac gccactcagt atatatagca cttccaaccc cgctttcctc gtggcgaggg

9001 cggccgacaa gttgcagaga cacgggagtg tcaagttgca ataaggccag tccgttatca

9061 gacgtgggaa cgtcggcacc gattcggtgc tttttttgcc gcggtcgcgc cgctcgctag

9121 caatatccca tagagcacgg tatcatcacc aactgggatg atatggagaa gatttggcat

9181 cacaccttct acaacgagtt gcgagtagct cctgaagaac atccagtatt gctgactgag

9241 gctcccttga atccaaagtc caatcgcgag aagatgactc agatcatgtt tgaaacattc

9301 gcttcgccag ctgtgtatgt tgccatccaa gctgttctgt ccctgtacgc ctccggtcgt

9361 actactggta ttgttctgga ttccggagat ggtgtctccc ataccgtccc aatctacgaa

9421 ggttatgctc tgccacatgc catcctccgt atggatttgg ctggtcgtga tctgaccgac

9481 tacctgatga agatcttgac cgaacgtgga tactctttca ccaccaccgc tgaacgtgaa

9541 atcgttcgtg acatcaagga gaagctgtgc tacgtcgctc tggacttcga gcaggaaatg

9601 caagccgctg ccgctacgtc ttcatccgag aagtcttatg aacttcccga tggccaagtc

9661 atcacaatcg gcaacgagcg tttccgtgct ccagaagccc ttttccagcc atccttcctg

9721 ggaatggaat caactggcat tcatgaaacg gtctacaact cgatcatgcg ttgcgatgtc

9781 gacatccgca aggatctcta tgctaacagc gtcttgtctg gtggtaccac catgtaccca

9841 ggtatttctt atatttaacc actcaactct gcatcatact caaaacctcc ctctattaca

9901 ggtattgctg atcgtatgca gaaggaaatc acttccctgg ctccatccac catcaagatc

9961 aagatcattg ccccaccgga acgtaaatac tccgtctgga tcggtggatc catcctggcc

10021 tcgctgtcta ccttccaagc tatgtggatc tccaagcagg aatacgacga aggtggccca

10081 ggaattgtcc accgcaagtg cttctaagcc gatcccgatt gtactgatta ccataagcga

10141 cattgccagt gaaagcgaca acagcagcat caaagtacat ttgtcatact gattcggcta

10201 ctaccaccat ccggaatcag cttgcatcga acatcaaatc acgttattca atgtatctgt

10261 catccagctc agacaagtcg gagcttttcc agtcgcgaaa atctgcgact ccagcggaaa

10321 gcaccgaacc acagagagga ctcgtatgaa agccagggaa gaaaccatca ttcaccttgc

10381 agcaaatagg aaaaaaaaac ggacatcttc aacaaacaaa agcccatgcg ctaacttggt

10441 ttaggagttt agtgtgacac catgaccccg ctgatgatct ttacttagca ccataaccac

10501 ctttatgcgt tcgttcatcc aaaatctaca ggatatcact gcagccgcga gaagaactcg

10561 tgaaccatcc tgttttcttt tttattatat tcttactttt aacttcaaat tattttcagt

10621 aataaaacgt ctcaaaataa taagttcata atgagtttaa ttttacggaa taagaacaac

10681 catttaagtt attaaatcct tagatttaat ggaattagat tgattatatg gaacccagac

10741 ttggtaaaaa ataaactcca cgttataatt ctttctgaga cttaaaattc tttcgggaaa

10801 gctgggagca attctcgcta aggcgtcgtc cacaaattat gaaacgcttt aattacgtga

10861 cggagtaggc tcaagcgtac gaatcataca aaaataatac aaatttttca tataaaaagc

10921 gttacgaagg gggaggtggt cgaaaattga caattgaccg gcccaatctt tgacattagt

10981 tttctttaat aattaaaatt atgcttgatt taaaattcat ctcgagtcat ctctgaattc

11041 ggttgttcaa ttgcatgggt ccttcgttag ttataattaa aaacgtgttt gaggacgtct

11101 cgattctaaa ctgttcttgg tcatggaaaa tcgatgttct tcttttattc tctcaagatt

11161 ttcaggctgt atattaaaac ttatattaag aactatgcta accacctcat caggaaccgt

11221 tgtaggtggc gtgggttttc ttggcaatcg actctcatga aaactacgag ctaaatattc

11281 aatatgttcc tcttgaccaa ctttattctg catttttttt gaacgaggtt tagagcaagc

11341 ttcaggaaac tgagacagga attttattaa aaatttaaat tttgaagaaa gttcagggtt

11401 aatagcatcc attttttgct ttgcaagttc ctcagcattc ttaacaaaag acgtctcttt

11461 tgacatgttt aaagtttaaa cctcctgtgt gaaattgtta tccgctcaca attccacaca

11521 ttatacgagc cggaagcata aagtgtaaag cctggggtgc ctaatgagtg agctaactca

11581 cattaattgc gttgcgctca ctgccaattg ctttccagtc gggaaacctg tcgtgccagc

11641 tgcattaatg aatcggccaa cgcgcgggga gaggcggttt gcgtattggg cgctcttccg

11701 cttcctcgct cactgactcg ctgcgctcgg tcgttcggct gcggcgagcg gtatcagctc

11761 actcaaaggc ggtaatacgg ttatccacag aatcagggga taacgcagga aagaacatgt

11821 gagcaaaagg ccagcaaaag gccaggaacc gtaaaaaggc cgcgttgctg gcgtttttcc

11881 ataggctccg cccccctgac gagcatcaca aaaatcgacg ctcaagtcag aggtggcgaa

11941 acccgacagg actataaaga taccaggcgt ttccccctgg aagctccctc gtgcgctctc

12001 ctgttccgac cctgccgctt accggatacc tgtccgcctt tctcccttcg ggaagcgtgg

12061 cgctttctca tagctcacgc tgtaggtatc tcagttcggt gtaggtcgtt cgctccaagc

12121 tgggctgtgt gcacgaaccc cccgttcagc ccgaccgctg cgccttatcc ggtaactatc

12181 gtcttgagtc caacccggta agacacgact tatcgccact ggcagcagcc actggtaaca

12241 ggattagcag agcgaggtat gtaggcggtg ctacagagtt cttgaagtgg tggcctaact

12301 acggctacac tagaaggaca gtatttggta tctgcgctct gctgaagcca gttaccttcg

12361 gaaaaagagt tggtagctct tgatccggca aacaaaccac cgctggtagc ggtggttttt

12421 ttgtttgcaa gcagcagatt acgcgcagaa aaaaaggatc tcaagaagat cctttgatct

12481 tttctacggg gtctgacgct cagtggaacg aaaactcacg ttaagggatt ttggtcatga

12541 gattatcaaa aaggatcttc acctagatcc ttttaaatta aaaatgaagt tttaaatcaa

12601 tc

//

LOCUS *190-perfect* 10395 bp ds-DNA circular 03-AUG-2021

DEFINITION .

SOURCE synthetic DNA construct

ORGANISM synthetic DNA construct

FEATURES Location/Qualifiers

misc_feature complement(205..309)

/label="AmpR Promoter"

primer_bind 700..720

/label="T7"

misc_feature 730..2730

/label="5'HomArm - AeAct4 (AAEL001951)"

misc_feature 769..1106

/label="Exon 1 - AeAct4 (AAEL001951)"

misc_feature 1107..2666

/label="Intron 1 - AeAct4 (AAEL001951)"

source 2724..2856

/label="Add_HA"

misc_feature 2857..4058

/label="Hr5IE1"

misc_feature 4103..4172

/label="adh Intron"

CDS 4208..4234

/label="NLS"

CDS 4208..4994

/label="Translation 4208-4994"

misc_feature 4244..4944

/label="AmCyan"

CDS 4956..4994

/label="NLS (2)"

CDS 4956..4994

/label="Translation 4956-4994"

3'UTR 5013..5794

/label="K10"

misc_feature 5795..5844

/label="attP50"

misc_feature 5945..6044

/label="AeU6-3 promoter_100bp"

misc_feature 6045..6064

/label="Act4-2 (Aegyp)"

misc_RNA 6065..6140

/label="gRNA scaffold_guide RNA scaffold for the CRISPR/Cas9 system"

misc_feature 6148..6181

/label="loxP"

misc_feature 6226..8225

/label="3'HomArm - AeAct4 (Seq.Corrected)"

misc_feature 6946..7005

/label="Intron 2 - AeAct4 (AAEL001951)"

misc_feature complement(8980..9568)

/label="pJet Ori"

ORIGIN

1 gctcatcatt ggaaaacgtt cttcggggcg aaaactctca aggatcttac cgctgttgag

61 atccagttcg atgtaaccca ctcgtgcacc caactgatct tcagcatctt ttactttcac

121 cagcgtttct gggtgagcaa aaacaggaag gcaaaatgcc gcaaaaaagg gaataagggc

181 gacacggaaa tgttgaatac tcatactctt cctttttcaa tattattgaa gcatttatca

241 gggttattgt ctcatgagcg gatacatatt tgaatgtatt tagaaaaata aacaaatagg

301 ggttccgcgc acatttcccc gaaaagtgcc acctgacgtc taagaaacca ttattatcat

361 gacattaacc tataaaaata ggcgtatcac gaggccgccc ctgcagccga attatattat

421 ttttgccaaa taatttttaa caaaagctct gaagtcttct tcatttaaat tcttagatga

481 tacttcatct ggaaaattgt cccaattagt agcatcacgc tgtgagtaag ttctaaacca

541 tttttttatt gttgtattat ctctaatctt actactcgat gagttttcgg tattatctct

601 atttttaact tggagcaggt tccattcatt gtttttttca tcatagtgaa taaaatcaac

661 tgctttaaca cttgtgcctg aacaccatat ccatccggcg taatacgact cactataggg

721 agagcggccg cacttccgag tataaaaccc cggtaaaccc aaggaatcac tcacaatcgg

781 attttgacgc tcgctctggt acagttcgat acggtctagt gaaaccgagg ataacgacga

841 aggtttttcc ccattgatcc aggtcggtgt ttatgattgg tggaaaaaga gctcgagaaa

901 agttccatcg aagccgttgg aaatgtgccg tcttcctgtg acgtcttgtg gatccagttc

961 cttgttcacg tctggtgatc gtgtaaaatg tgctgtcttg tggcgtcata tgtgttccag

1021 atccagtgat tacgatccga tgtgatgttg atcccttgtg aacgtcttat cctgttccgt

1081 gtgcaccatg cataatgtcg tattacgtaa gttctgaagt gaaacagaag agtgaattga

1141 aagttttttt tattcaacat caacctaaat atggacttta ctttccaaga aaattatgcc

1201 tgatcaactg tggatagtta caaaaaaaaa aggtttatta attaaatttt atgattacat

1261 aatgtgttga aaagaacaac tgaaatttta gaagaagatc ttttcgtgca tcaggctttg

1321 ccaattaatt gatgataaat tatcatagca aattaacgta gagactaaaa ggtatatcgt

1381 caaatagggc ttcttttgac actattttgg cattcttgct ctttgagaac ttgcaaccct

1441 aaaatgggat cttcatcagc ctagtggtta gattcagcag ctacaaagca aagccatgct

1501 gaagggttcg attcccggtc gtttcaggat cttttcgtaa ttgaaatatc cttgactgcc

1561 ctgagtatca ttgtgcttgc catttacgaa tatacatatt acgatatacg aatgagaaaa

1621 tgacaacttt ggaaaataaa gctctcaatg tttcaataag aaataaatac tacatcagta

1681 ttgaaggcta ataacaatta cagattagaa cctttaaaca tcatttctgc aacaggctgg

1741 ataaagtaca gttggaggat taaattatgc gattttgcaa ttttttccga ttaaattcat

1801 atttattcct ggtttggttt ttacaaaaaa tatttttaca tgacgtttga ccccgattcc

1861 ctcaactttg attgttatat ttttttttgg acaggttgag tttgtgggtt ttttcctagt

1921 gttgctttgc cttatgggct ctggttattt aaaattaaaa tttgacaatc ttactacaca

1981 ctccgaaaaa atcatgcgat tttacgtctt ttggatgcac ataaaagaag cgagccaaat

2041 gaggtgaatt tgtgtcacat tttaaatacg atggtgtctg attcgggaaa tgtcaatgat

2101 agtgtcattc aatcataatg tgaattacgt ccgcagtaat tttcattatt tttaagagtg

2161 tactactatt tacactacaa aaattttgat accccagggg ggaacgaggt cccggatgtc

2221 cagctggcca gattgttggc aacgagccct gtacctattg atcgagtcac caaagcactc

2281 ctcaagtgtt ttaatctcga ccagacggtg gacctcggtt gttctcattc tcggagggcg

2341 atttcgcaat cattagtacc aaccacatgt cgaagtcggg agatgttata aaattataac

2401 caattattca aaaaatgaca tcattcaatt tgaacaaacg ttcgatagaa attatatatg

2461 atttcacatg atattaaact acgaagaaaa ttttacataa ggaagtggta taaaacgtaa

2521 tatgcttaat aaaaacttta acccttttgg gaggataata ttcagaagtt ctgattcaga

2581 accatctctc atgttatgtt cgttttttgt tgcttgtcct ttatatgcca catgaacaat

2641 aacaccaata tctatcccat ttccaggacc taacggacct tgaagcggcg ccaaaatgtg

2701 tgacgatgat gctggagcac tagtcattga caacggatcc ggcatgtgta aggccggttt

2761 cgctggtgat gatgccccac gtgccgtctt cccgtccatt gtcggccgcc ctcgccacca

2821 gggtgtgatg gtcggtatgg gtcaaaaaga tgcctagctt tacgagtaga attctacgcg

2881 taaaacacaa tcaagtatga gtcataatct gatgtcatgt tttgtacacg gctcataacc

2941 gaactggctt tacgagtaga attctacttg taatgcacga tcagtggatg atgtcatttg

3001 tttttcaaat cgagatgatg tcatgttttg cacacggctc ataaactcgc tttacgagta

3061 gaattctacg tgtaacgcac gatcgattga tgagtcattt gttttgcaat atgatatcat

3121 acaatatgac tcatttgttt ttcaaaaccg aacttgattt acgggtagaa ttctacttgt

3181 aaagcacaat caaaaagatg atgtcatttg tttttcaaaa ctgaactcgc tttacgagta

3241 gaattctacg tgtaaaacac aatcaagaaa tgatgtcatt tgttataaaa ataaaagctg

3301 atgtcatgtt ttgcacatgg ctcataacta aactcgcttt acgggtagaa ttctacgcgt

3361 aaaacatgat tgataattaa ataattcatt tgcaagctat acgttaaatc aaacggacgc

3421 tcgaggttgc acaacactat tatcgatttg cagttcggga cataaatgtt taaatatatc

3481 gatgtctttg tgatgcgcgc gacatttttg taggttattg ataaaatgaa cggatacgtt

3541 gcccgacatt atcattaaat ccttggcgta gaatttgtcg ggtccattgt ccgtgtgcgc

3601 tagtagcatg cccgtaacgg acctcgtact tttggcttca aaggttttgc gcacagacaa

3661 aatgtgccac acttgcagct ctgcatgtgt gcgcgttacc acaaatccca acggcgcagt

3721 gtacttgttg tatgcaaata aatctcgata aaggcgcggc gcgcgaatgc agctgatcac

3781 gtacgctcct cgtgttccgt tcaaggacgg tgttatcgac ctcagattaa tgtttatcgg

3841 ccgactgttt tcgtatccgc tcaccaaacg cgtttttgca ttaacattgt atgtcggcgg

3901 atgttctata tctaatttga ataaataaac gataaccgcg ttggttttag agggcataat

3961 aaaagaaata ttgttatcgt gttcgccatt agggcagtat aaattgacgt tcatgttgga

4021 tattgtttca gttgcaagtt gacactggcg gcgacaagca attggtaccc gggtaggatc

4081 ctagtgaatt cctaatctgg cggtaagttg atcaaaggaa acgcaaagtt ttcaagaaaa

4141 aacaaaacta atttgattta taacaccttt agaaagcgaa gttgagattc aggccaccat

4201 gggagatccc accccaccca agaagaagcg caaagctagc gttatggccc tgtccaacaa

4261 gttcatcggc gacgacatga agatgaccta ccacatggac ggctgcgtga acggccacta

4321 cttcaccgtg aagggcgagg gcagcggcaa gccctacgag ggcacccaga cctccacctt

4381 caaagtcaca atggccaacg gcggccccct ggccttctcc ttcgacatcc tgtccaccgt

4441 gttcatgtac ggcaaccgct gcttcaccgc ctaccccacc agcatgcccg actacttcaa

4501 gcaggccttc cccgacggca tgtcctacga gagaaccttc acctacgagg acggcggcgt

4561 ggccaccgcc agctgggaga tcagcctgaa gggcaactgc ttcgagcaca agtccacctt

4621 ccacggcgtg aacttccccg ccgacggccc cgtgatggcc aagaagacca ccggctggga

4681 cccctccttc gagaagatga ccgtgtgcga cggcatcttg aagggcgacg tgaccgcctt

4741 cctgatgctg caaggcggcg gcaactacag atgccagttc cacacctcct acaagaccaa

4801 gaagcccgtg accatgcccc ccaaccacgt ggtggagcac cgcatcgcca gaaccgacct

4861 ggacaagggc ggcaacagcg tgcagctgac cgagcacgcc gtggcccaca tcacctccgt

4921 ggtgcccttc tccggactcc gctcccagat ctcccgaccc aagaaaaagc ggaaggtgga

4981 ggacccgtaa gatccaccgg atctagataa ctggagcttg ataacattat acctaaaccc

5041 atggtcaaga gtaaacattt ctgcctttga agttgagaac acaattaagc atcccctggt

5101 taaacctgac attcatactt gttaatagcg ccataaacat agcaccaatt tcgaagaaat

5161 cagttaaaag caattagcaa ttagcaatta gcaataactc tgctgacttc aaaacgagaa

5221 gagttgcaag tatttgtaag gcacagttta tagaccaccg acggctcatt agggctcgtc

5281 atgtaactaa gcgcggtgaa acccaattga acatatagtg gaattattat tatcaatggg

5341 gaagatttaa ccctcaggta gcaaagtaat ttaattgcaa atagagagtc ctaagactaa

5401 ataatatatt taaaaatctg gccctttgac cttgcttgtc aggtgcattt gggttcaatc

5461 gtaagttgct tctatataaa cactttcccc atccccgcaa taatgaagaa taccgcagaa

5521 taaagagaga tttgcaacaa aaaataaagg cattgcgaaa actttttatg ggggatcatt

5581 acactcgggc ctacggttac aattcccagc cacttaagcg acaagtttgg ccaacaatcc

5641 atctaatagc taatagcgca atcactggta atcgcaagag tatataggca atagaaccca

5701 tggatttgac caaaggtaac cgagacaatg gagaagcaag aggatttcaa actgaacacc

5761 cacagtactg tgtactacca ctggcgcgtt tgggagtagt gccccaactg gggtaacctt

5821 tgagttctct cagttggggg cgtactctac ccgttcaggc agcattcatc gaaaagccct

5881 atctgctcgc acacatttac aaaatgctga ttgcgttgtg tgctgaatgg gtcactcgtc

5941 cgtcactgct tgctgtgtac actgtacagt tacgcagtct gtgcatcgct agaatcatat

6001 ttacggaaga gtattatata tacccgatgc gttgctcttc gattggtcaa aaagatgcct

6061 acgtgtttta gagctagaaa tagcaagtta aaataaggct agtccgttat caacttgaaa

6121 aagtggcacc gagtcggtgc tttttttata acttcgtata atgtatgcta tacgaagtta

6181 tcgtcggtga tgaagcccaa tccaagcgag gtatcctcac cctgaaatat cccatagagc

6241 acggtatcat caccaactgg gatgatatgg agaagatttg gcatcacacc ttctacaacg

6301 agttgcgagt agctcctgaa gaacatccag tattgctgac tgaggctccc ttgaatccaa

6361 agtccaatcg cgagaagatg actcagatca tgtttgaaac attcgcttcg ccagctgtgt

6421 atgttgccat ccaagctgtt ctgtccctgt acgcctccgg tcgtactact ggtattgttc

6481 tggattccgg agatggtgtc tcccataccg tcccaatcta cgaaggttat gctctgccac

6541 atgccatcct ccgtatggat ttggctggtc gtgatctgac cgactacctg atgaagatct

6601 tgaccgaacg tggatactct ttcaccacca ccgctgaacg tgaaatcgtt cgtgacatca

6661 aggagaagct gtgctacgtc gctctggact tcgagcagga aatgcaagcc gctgccgcta

6721 cgtcttcatc cgagaagtct tatgaacttc ccgatggcca agtcatcaca atcggcaacg

6781 agcgtttccg tgctccagaa gcccttttcc agccatcctt cctgggaatg gaatcaactg

6841 gcattcatga aacggtctac aactcgatca tgcgttgcga tgtcgacatc cgcaaggatc

6901 tctatgctaa cagcgtcttg tctggtggta ccaccatgta cccaggtatt tcttatattt

6961 aaccaatcaa ctctgcatca tactcaaaac ctccctctat tacaggtatt gctgatcgta

7021 tgcagaagga aatcacttcc ctggctccat ccaccatcaa gatcaagatc attgccccac

7081 cggaacgtaa atactccgtc tggatcggtg gatccatcct ggcctctctg tctaccttcc

7141 aagctatgtg gatctccaag caggaatacg acgaaggtgg cccaggaatt gtccaccgca

7201 agtgcttcta agccgatccc gattgtactg attaccataa gcgacattgc cagtgaaagc

7261 gacaacagca gcatcaaagt acatttgtca tactgattcg gctactacca ccatccggaa

7321 tcagcttgca tcgaacatca aatcacgtta ttcaatgtat ctgtcatcca gctcagacaa

7381 gtcggagctt ttccagtcgc gaaaatctgc gactccagcg gaaagcgccg aaccacagag

7441 aggactcgta tgaaagccag ggaagaaacc atcattcacc ttgcagcaaa taggaaaaaa

7501 aaacggacat cttcaacaaa caaaagccca tgcgctaact tggtttagga gtttagtgtg

7561 acaccatgac cccgctgatg atctttactt agcaccataa ccacctttat gcgttcgttc

7621 atccaaaatc tacaggatat cactgcagcc gcgagaagaa ctcgtgaacc atcctgtttt

7681 cttttttatt atattcttac ttttaacttc aaattatttt cagtaataaa acgtctcaaa

7741 ataataagtt cataatgagt ttaattttac ggaataagaa caaccattta agttattaaa

7801 tccttagatt taatggaatt agattgatta tatggaaccc agacttggta aaaaataaac

7861 tccacgttat aattctttct gagacttaaa attctttcgg gaaagctggg agcaattctc

7921 gctaaggcgt cgtccacaaa ttatgaaacg ctttaattac gtgacggagt aggctcaagc

7981 gtacgaatca tacaaaaata atacaaattt ttcatataaa aagcgttacg aagggggagg

8041 tggtcgaaaa ttgacaattg accggcccaa tctttgacat tagttttctt taataattaa

8101 aattatgctt gatttaaaat tcatctcgag tcatctctga attcggttgt tcaattgcat

8161 gggtccttcg ttagttataa ttaaaaacgt gtttgaggac gtctcgattc taaactgttc

8221 ttggtcatgg aaaatcgatg ttcttctttt attctctcaa gattttcagg ctgtatatta

8281 aaacttatat taagaactat gctaaccacc tcatcaggaa ccgttgtagg tggcgtgggt

8341 tttcttggca atcgactctc atgaaaacta cgagctaaat attcaatatg ttcctcttga

8401 ccaactttat tctgcatttt ttttgaacga ggtttagagc aagcttcagg aaactgagac

8461 aggaatttta ttaaaaattt aaattttgaa gaaagttcag ggttaatagc atccattttt

8521 tgctttgcaa gttcctcagc attcttaaca aaagacgtct cttttgacat gtttaaagtt

8581 taaacctcct gtgtgaaatt attatccgct cataattcca cacattatac gagccggaag

8641 cataaagtgt aaagcctggg gtgcctaatg agtgagctaa ctcacattaa ttgcgttgcg

8701 ctcactgcca attgctttcc agtcgggaaa cctgtcgtgc cagctgcatt aatgaatcgg

8761 ccaacgcgcg gggagaggcg gtttgcgtat tgggcgctct tccgcttcct cgctcactga

8821 ctcgctgcgc tcggtcgttc ggctgcggcg agcggtatca gctcactcaa aggcggtaat

8881 acggttatcc acagaatcag gggataacgc aggaaagaac atgtgagcaa aaggccagca

8941 aaaggccagg aaccgtaaaa aggccgcgtt gctggcgttt ttccataggc tccgcccccc

9001 tgacgagcat cacaaaaatc gacgctcaag tcagaggtgg cgaaacccga caggactata

9061 aagataccag gcgtttcccc ctggaagctc cctcgtgcgc tctcctgttc cgaccctgcc

9121 gcttaccgga tacctgtccg cctttctccc ttcgggaagc gtggcgcttt ctcatagctc

9181 acgctgtagg tatctcagtt cggtgtaggt cgttcgctcc aagctgggct gtgtgcacga

9241 accccccgtt cagcccgacc gctgcgcctt atccggtaac tatcgtcttg agtccaaccc

9301 ggtaagacac gacttatcgc cactggcagc agccactggt aacaggatta gcagagcgag

9361 gtatgtaggc ggtgctacag agttcttgaa gtggtggcct aactacggct acactagaag

9421 aacagtattt ggtatctgcg ctctgctgaa gccagttacc ttcggaaaaa gagttggtag

9481 ctcttgatcc ggcaaacaaa ccaccgctgg tagcggtggt ttttttgttt gcaagcagca

9541 gattacgcgc agaaaaaaag gatctcaaga agatcctttg atcttttcta cggggtctga

9601 cgctcagtgg aacgaaaact cacgttaagg gattttggtc atgagattat caaaaaggat

9661 cttcacctag atccttttaa attaaaaatg aagttttaaa tcaatctaaa gtatatatga

9721 gtaaacttgg tctgacagtt accaatgctt aatcagtgag gcacctatct cagcgatctg

9781 tctatttcgt tcatccatag ttgcctgact ccccgtcgtg tagataacta cgatacggga

9841 gggcttacca tctggcccca gtgctgcaat gataccgcgc gacccacgct caccggctcc

9901 agatttatca gcaataaacc agccagccgg aagggccgag cgcagaagtg gtcctgcaac

9961 tttatccgcc tccatccagt ctattaattg ttgccgggaa gctagagtaa gtagttcgcc

10021 agttaatagt ttgcgcaacg ttgttgccat tgctacaggc atcgtggtgt cacgctcgtc

10081 gtttggtatg gcttcattca gctccggttc ccaacgatca aggcgagtta catgatcccc

10141 catgttgtgc aaaaaagcgg ttagctcctt cggtcctccg atcgttgtca gaagtaagtt

10201 ggccgcagtg ttatcactca tggttatggc agcactgcat aattctctta ctgtcatgcc

10261 atccgtaaga tgcttttctg tgactggtga gtactcaacc aagtcattct gagaatagtg

10321 tatgcggcga ccgagttgct cttgcccggc gtcaatacgg gataataccg cgccacatag

10381 cagaacttta aaagt

//

LOCUS *64+234-perfect* 11923 bp ds-DNA circular 03-AUG-2021

DEFINITION .

FEATURES Location/Qualifiers

CDS complement(33..893)

/label="AmpR - BsaI Recoded"

misc_feature complement(894..998)

/label="AmpR Promoter"

CDS complement(1101..1418)

/label="Eco47I/T7 (CUT) (2)"

misc_feature 1419..3411

/label="5'HomArm - AeAct4 (AAEL001951) (7bp SHORT)"

misc_feature 1458..1795

/label="Exon 1 - AeAct4 (AAEL001951)"

misc_feature 1796..3354

/label="Intron 1 - AeAct4 (AAEL001951)"

5'UTR 3355..3383

/label="5'UTR (2) - AeAct4 (AAEL001951)"

misc_feature 3415..3464

/label="attP50"

misc_feature 3474..4675

/label="Hr5IE1"

misc_feature 4720..4789

/label="adh Intron"

CDS 4825..4851

/label="NLS"

misc_feature 4864..5564

/label="AmCyan"

CDS 5576..5614

/label="NLS (2)"

3'UTR 5633..6414

/label="K10"

misc_feature 6431..6630

/label="U6-2"

misc_feature 6631..6650

/label="Act4-1 (Aegyp)"

misc_RNA 6651..6726

/label="gRNA scaffold_guide RNA scaffold for the CRISPR/Cas9 system"

terminator 6727..6733

/label="pol_III\terminator"

misc_feature 6734..7303

/label="Putative 7SK promoter"

misc_feature 7304..7323

/label="Act4-2 (Aegyp)"

misc_feature 7324..7399

/label="sgRNA backbone 22"

terminator 7400..7406

/label="pol_III\terminator"

misc_feature 7407..7606

/label="U6-3"

misc_feature 7607..7626

/label="Act4-3 (Aegyp)"

misc_feature 7627..7702

/label="sgRNA backbone 23"

terminator 7703..7709

/label="pol_III\terminator"

misc_feature 7710..8309

/label="Ae albopictus 7SK"

misc_feature 8310..8329

/label="Act4-8 (Aegyp)"

misc_feature 8330..8411

/label="sgRNA backbone 15"

terminator 8412..8418

/label="pol_III\terminator"

misc_feature 8443..10442

/label="3'HomArm - AeAct4 (Seq.Corrected)"

misc_feature 9163..9222

/label="Intron 2 - AeAct4 (AAEL001951)"

misc_feature 9223..10083

/label="Exon 3 - AeAct4 (AAEL001951)"

CDS complement(10443..10787)

/label="Eco47I/T7 (CUT) (1)"

misc_feature complement(10816..10832)

/label="Lac Operator"

misc_feature complement(10840..10870)

/label="Lac UV5"

misc_feature complement(11197..11785)

/label="pJet Ori"

ORIGIN

1 taaagtatat atgagtaaac ttggtctgac agttaccaat gcttaatcag tgaggcacct

61 atctcagcga tctgtctatt tcgttcatcc atagttgcct gactccccgt cgtgtagata

121 actacgatac gggagggctt accatctggc cccagtgctg caatgatacc gcgcgaccca

181 cgctcaccgg ctccagattt atcagcaata aaccagccag ccggaagggc cgagcgcaga

241 agtggtcctg caactttatc cgcctccatc cagtctatta attgttgccg ggaagctaga

301 gtaagtagtt cgccagttaa tagtttgcgc aacgttgttg ccattgctac aggcatcgtg

361 gtgtcacgct cgtcgtttgg tatggcttca ttcagctccg gttcccaacg atcaaggcga

421 gttacatgat cccccatgtt gtgcaaaaaa gcggttagct ccttcggtcc tccgatcgtt

481 gtcagaagta agttggccgc agtgttatca ctcatggtta tggcagcact gcataattct

541 cttactgtca tgccatccgt aagatgcttt tctgtgactg gtgagtactc aaccaagtca

601 ttctgagaat agtgtatgcg gcgaccgagt tgctcttgcc cggcgtcaat acgggataat

661 accgcgccac atagcagaac tttaaaagtg ctcatcattg gaaaacgttc ttcggggcga

721 aaactctcaa ggatcttacc gctgttgaga tccagttcga tgtaacccac tcgtgcaccc

781 aactgatctt cagcatcttt tactttcacc agcgtttctg ggtgagcaaa aacaggaagg

841 caaaatgccg caaaaaaggg aataagggcg acacggaaat gttgaatact catactcttc

901 ctttttcaat attattgaag catttatcag ggttattgtc tcatgagcgg atacatattt

961 gaatgtattt agaaaaataa acaaataggg gttccgcgca catttccccg aaaagtgcca

1021 cctgacgtct aagaaaccat tattatcatg acattaacct ataaaaatag gcgtatcacg

1081 aggccgcccc tgcagccgaa ttatattatt tttgccaaat aatttttaac aaaagctctg

1141 aagtcttctt catttaaatt cttagatgat acttcatctg gaaaattgtc ccaattagta

1201 gcatcacgct gtgagtaagt tctaaaccat ttttttattg ttgtattatc tctaatctta

1261 ctactcgatg agttttcggt attatctcta tttttaactt ggagcaggtt ccattcattg

1321 tttttttcat catagtgaat aaaatcaact gctttaacac ttgtgcctga acaccatatc

1381 catccggcgt aatacgactc actataggga gagcggccgc acttccgagt ataaaacccc

1441 ggtaaaccca aggaatcact cacaatcgga ttttgacgct cgctctggta cagttcgata

1501 cggtctagtg aaaccgagga taacgacgaa ggtttttccc cattgatcca ggtcggtgtt

1561 tatgattggt ggaaaaagag ctcgagaaaa gttccatcga agccgttgga aatgtgccgt

1621 cttcctgtga cgtcttgtgg atccagttcc ttgttcacgt ctggtgatcg tgtaaaatgt

1681 gctgtcttgt ggcgtcatat gtgttccaga tccagtgatt acgatccgat gtgatgttga

1741 tcccttgtga acgtcttatc ctgttccgtg tgcaccatgc ataatgtcgt attacgtaag

1801 ttctgaagtg aaacagaaga gtgaattgaa agttttttta ttcaacatca acctaaatat

1861 ggactttact ttccaagaaa attatgcctg atcaactgtg gatagttaca aaaaaaaaag

1921 gtttattaat taaattttat gattacataa tgtgttgaaa agaacaactg aaattttaga

1981 agaagatctt ttcgtgcatc aggctttgcc aattaattga tgataaatta tcatagcaaa

2041 ttaacgtaga gactaaaagg tatatcgtca aatagggctt cttttgacac tattttggca

2101 ttcttgctct ttgagaactt gcaaccctaa aatgggatct tcatcagcct agtggttaga

2161 ttcagcagct acaaagcaaa accatgctga agggttcgat tcccggtcgt ttcaggatct

2221 tttcgtaatt gaaatatcct tgactaccct aagtatcatt gtgcttgcca tttacgaata

2281 tacatattac gatatacgaa tgagaaaatg acaactttgg aaaataaagc tctcaatgtt

2341 tcaataagaa ataaatacta catcagtatt gaaggctaat aacaattaca gattagaacc

2401 tttaaacatc atttctgcaa caggctggat aaagtacagt tggaggatta aattatgcga

2461 ttttgcaatt ttttccgatt aaattcatat ttattcctgg tttggttttt acaaaaaata

2521 tttttacatg acgtttgacc ccgattccct caactttgat tgttatattt ttttttggac

2581 aggttgagtt tgtgggtttt ttcctagtgt tgctttgctt tatgggctct ggttatttaa

2641 aattaaaatt tgacaatctt actacacact ccgaaaaaat catgcgattt tacgtctttt

2701 ggatgcacat aaaagaagcg agccaaatga ggtgaatttg tgtcacattt taaatacgat

2761 ggtgtctgat tcgggaaatg tcaatgatag tgtcattcaa tcataatgtg aattacgtcc

2821 gcagtaattt tcattatttt taagagtgta ctactattta cactacaaaa attttgatac

2881 cccagggggg aacgaggtcc cggatgtcca gctggccaga ttgttggcaa cgagccctgt

2941 acctattgat cgagtcacca aagcactcct caagtgtttt aatctcgacc agacggtgga

3001 cctcggttgt tctcattctc ggagggcgat ttcgcaatca ttagtaccaa ccacatgtcg

3061 aagtcgggag atgttataaa attataacca attattcaaa aaatgacatc attcaatttg

3121 aacaaacgtt cgatagaaat tatatatgat ttcacatgat attaaactac gaagaaaatt

3181 ttacataagg aagtggtata aaacgtaata tgcttaataa aaactttaac ccttttggga

3241 ggataatatt cagaagttct gattcagaac catctctcat gttatgttcg ttttttgttg

3301 cttgtccttt atatgccaca tgaacaataa caccaatatc tatcccattt ccaggaccta

3361 acggaccttg aagcggcgcc aaaatgtgtg acgatgatgc tggagcacta gccaagtagt

3421 gccccaactg gggtaacctt tgagttctct cagttggggg cgtaggtcga caagctttac

3481 gagtagaatt ctacgcgtaa aacacaatca agtatgagtc ataatctgat gtcatgtttt

3541 gtacacggct cataaccgaa ctggctttac gagtagaatt ctacttgtaa tgcacgatca

3601 gtggatgatg tcatttgttt ttcaaatcga gatgatgtca tgttttgcac acggctcata

3661 aactcgcttt acgagtagaa ttctacgtgt aacgcacgat cgattgatga gtcatttgtt

3721 ttgcaatatg atatcataca atatgactca tttgtttttc aaaaccgaac ttgatttacg

3781 ggtagaattc tacttgtaaa gcacaatcaa aaagatgatg tcatttgttt ttcaaaactg

3841 aactcgcttt acgagtagaa ttctacgtgt aaaacacaat caagaaatga tgtcatttgt

3901 tataaaaata aaagctgatg tcatgttttg cacatggctc ataactaaac tcgctttacg

3961 ggtagaattc tacgcgtaaa acatgattga taattaaata attcatttgc aagctatacg

4021 ttaaatcaaa cggacgctcg aggttgcaca acactattat cgatttgcag ttcgggacat

4081 aaatgtttaa atatatcgat gtctttgtga tgcgcgcgac atttttgtag gttattgata

4141 aaatgaacgg atacgttgcc cgacattatc attaaatcct tggcgtagaa tttgtcgggt

4201 ccattgtccg tgtgcgctag tagcatgccc gtaacggacc tcgtactttt ggcttcaaag

4261 gttttgcgca cagacaaaat gtgccacact tgcagctctg catgtgtgcg cgttaccaca

4321 aatcccaacg gcgcagtgta cttgttgtat gcaaataaat ctcgataaag gcgcggcgcg

4381 cgaatgcagc tgatcacgta cgctcctcgt gttccgttca aggacggtgt tatcgacctc

4441 agattaatgt ttatcggccg actgttttcg tatccgctca ccaaacgcgt ttttgcatta

4501 acattgtatg tcggcggatg ttctatatct aatttgaata aataaacgat aaccgcgttg

4561 gttttagagg gcataataaa agaaatattg ttatcgtgtt cgccattagg gcagtataaa

4621 ttgacgttca tgttggatat tgtttcagtt gcaagttgac actggcggcg acaagcaatt

4681 ggtacccggg taggatccta gtgaattcct aatctggcgg taagttgatc aaaggaaacg

4741 caaagttttc aagaaaaaac aaaactaatt tgatttataa cacctttaga aagcgaagtt

4801 gagattcagg ccaccatggg agatcccacc ccacccaaga agaagcgcaa accggctagc

4861 gttatggccc tgtccaacaa gttcatcggc gacgacatga agatgaccta ccacatggac

4921 ggctgcgtga acggccacta cttcaccgtg aagggcgagg gcagcggcaa gccctacgag

4981 ggcacccaga cctccacctt caaagtcaca atggccaacg gcggccccct ggccttctcc

5041 ttcgacatcc tgtccaccgt gttcatgtac ggcaaccgct gcttcaccgc ctaccccacc

5101 agcatgcccg actacttcaa gcaggccttc cccgacggca tgtcctacga gagaaccttc

5161 acctacgagg acggcggcgt ggccaccgcc agctgggaga tcagcctgaa gggcaactgc

5221 ttcgagcaca agtccacctt ccacggcgtg aacttccccg ccgacggccc cgtgatggcc

5281 aagaagacca ccggctggga cccctccttc gagaagatga ccgtgtgcga cggcatcttg

5341 aagggcgacg tgaccgcctt cctgatgctg caaggcggcg gcaactacag atgccagttc

5401 cacacctcct acaagaccaa gaagcccgtg accatgcccc ccaaccacgt ggtggagcac

5461 cgcatcgcca gaaccgacct ggacaagggc ggcaacagcg tgcagctgac cgagcacgcc

5521 gtggcccaca tcacctccgt ggtgcccttc tccggactcc gctcccagat ctcccgaccc

5581 aagaaaaagc ggaaggtgga ggacccgtaa gatccaccgg atctagataa ctggagcttg

5641 ataacattat acctaaaccc atggtcaaga gtaaacattt ctgcctttga agttgagaac

5701 acaattaagc atcccctggt taaacctgac attcatactt gttaatagcg ccataaacat

5761 agcaccaatt tcgaagaaat cagttaaaag caattagcaa ttagcaatta gcaataactc

5821 tgctgacttc aaaacgagaa gagttgcaag tatttgtaag gcacagttta tagaccaccg

5881 acggctcatt agggctcgtc atgtaactaa gcgcggtgaa acccaattga acatatagtg

5941 gaattattat tatcaatggg gaagatttaa ccctcaggta gcaaagtaat ttaattgcaa

6001 atagagagtc ctaagactaa ataatatatt taaaaatctg gccctttgac cttgcttgtc

6061 aggtgcattt gggttcaatc gtaagttgct tctatataaa cactttcccc atccccgcaa

6121 taatgaagaa taccgcagaa taaagagaga tttgcaacaa aaaataaagg cattgcgaaa

6181 actttttatg ggggatcatt acactcgggc ctacggttac aattcccagc cacttaagcg

6241 acaagtttgg ccaacaatcc atctaatagc taatagcgca atcactggta atcgcaagag

6301 tatataggca atagaaccca tggatttgac caaaggtaac cgagacaatg gagaagcaag

6361 aggatttcaa actgaacacc cacagtactg tgtactacca ctggcgcgtt tgggtacggt

6421 gaggccggcc agttgggacg tttgactttt tgtaggtaga caaaaactaa actttttttc

6481 gcttctctat gtgtgccccc ccgggtagcg tatcgttccg attgtggtgc gaacgaatga

6541 aatcgcccat cgagttgata cgtccatcca tcgctagaac cgcgttcgct gtagaagact

6601 atataagagc agaggcaaga gtagtgaaat ggagcactag tcattgacaa gttttagagc

6661 tagaaatagc aagttaaaat aaggctagtc cgttatcaac ttgaaaaagt ggcaccgagt

6721 cggtgctttt tttatgctcc attctccgcc acttgttgat gcggacccta accacgtggt

6781 cgctcctctg ctcaccggag cacgtttcat acagcctgac gacgacgagc aatcagaggt

6841 atggtgagca tgcgcatgga gagtggacag cagtgcaccc taaaatcaat tcacacatca

6901 tgtgtcaata gctgtgtcaa tgttgcacag ccttttctta ttaaatttac tccttttgtg

6961 accatttctc tttcatccac cgttatttta atgagttttg tgttccggtg gacgaacgtt

7021 cacacaaaaa atgtgtaaat cttaatcaac cagaacacaa agtatagtga aaaaattaaa

7081 gtgtgtggct tttatacatc ctaactgtaa attattttta gagtgcgtgc gatcgttctc

7141 tcgaaccacg ctctccgcta cacattcgca gcgaatggcg tgaatggatg aaagaacaaa

7201 ctaaagttta tttttagatt cgtctcaaaa caactgctgt gcatcgctag aaccaagaaa

7261 tacgccactc agtatatata gcacttccaa ccccgctttc ctcggtcaaa aagatgccta

7321 cgtgttcgag aggacgagag tccaagttcg aataaggcca gtccgttatc acagggagac

7381 ctgggcaccg agtcggtgct ttttttctct acccgttcag gcagcattca tcgaaaagcc

7441 ctatctgctc gcacacattt acaaaatgct gattgcgttg tgtgctgaat gggtcactcg

7501 tccgtcactg cttgctgtgt acactgtaca gttacgcagt ctgtgcatcg ctagaatcat

7561 atttacggaa gagtattata tatacccgat gcgttgctct tcgattgtgc tctatgggat

7621 atttcagttc cagagtcggg aacgacaagt tggaataagg caagtccgtt atcatgccgg

7681 aaggcaggca ccgattcggt gctttttttt agcatagtca gtcagtgaat aagcacccaa

7741 gccaagcgac catccaccga acaactgagg agggagattg ttacaagcac agctgattcg

7801 atctcgctct cggtaccgca tggcttgcga ggcaagagca tcaagctacg agcaggcaaa

7861 acaacacccc tcataaaccg taaacatacc cttacgctta ccccatccat tccctaccgt

7921 agcgaccagc tcattatggg aaacccgaaa cagattttat gttatgcttc tttctctgcc

7981 acttgttgat gccgcatcaa gagaccacgc ggtcgctcga ccacgttcca tgcggcacag

8041 gtagaggagg taggcaatcg agtgcgcgcc agaggcatgg tgagcatgcg cacagagaga

8101 cgaccgcacc atgtgaggcg atcgttctct ccgttctgaa agctctcctc gctccgtcgt

8161 tttgaattaa tttgtatgag taaaggtagg caaaagttat ttttagccac tcgactcgag

8221 acgttgaatt catagcaact gccatccatc gctaaaaccg aaattttcgc agtctactat

8281 atatacgact tccaccaccg gatatcttct cacaccctgg tggcgagggg tgttagaggg

8341 atagagatat cccaagttaa cataaggcta gtccgttatc actgcaggaa tgcagggcac

8401 cgagtcggtg ctttttttgc cgcggtcgcg ccgctcgcta gcaatatccc atagagcacg

8461 gtatcatcac caactgggat gatatggaga agatttggca tcacaccttc tacaacgagt

8521 tgcgagtagc tcctgaagaa catccagtat tgctgactga ggctcccttg aatccaaagt

8581 ccaatcgcga gaagatgact cagatcatgt ttgaaacatt cgcttcgcca gctgtgtatg

8641 ttgccatcca agctgttctg tccctgtacg cctccggtcg tactactggt attgttctgg

8701 attccggaga tggtgtctcc cataccgtcc caatctacga aggttatgct ctgccacatg

8761 ccatcctccg tatggatttg gctggtcgtg atctgaccga ctacctgatg aagatcttga

8821 ccgaacgtgg atactctttc accaccaccg ctgaacgtga aatcgttcgt gacatcaagg

8881 agaagctgtg ctacgtcgct ctggacttcg agcaggaaat gcaagccgct gccgctacgt

8941 cttcatccga gaagtcttat gaacttcccg atggccaagt catcacaatc ggcaacgagc

9001 gtttccgtgc tccagaagcc cttttccagc catccttcct gggaatggaa tcaactggca

9061 ttcatgaaac ggtctacaac tcgatcatgc gttgcgatgt cgacatccgc aaggatctct

9121 atgctaacag cgtcttgtct ggtggtacca ccatgtaccc aggtatttct tatatttaac

9181 cactcaactc tgcatcatac tcaaaacctc cctctattac aggtattgct gatcgtatgc

9241 agaaggaaat cacttccctg gctccatcca ccatcaagat caagatcatt gccccaccgg

9301 aacgtaaata ctccgtctgg atcggtggat ccatcctggc ctcgctgtct accttccaag

9361 ctatgtggat ctccaagcag gaatacgacg aaggtggccc aggaattgtc caccgcaagt

9421 gcttctaagc cgatcccgat tgtactgatt accataagcg acattgccag tgaaagcgac

9481 aacagcagca tcaaagtaca tttgtcatac tgattcggct actaccacca tccggaatca

9541 gcttgcatcg aacatcaaat cacgttattc aatgtatctg tcatccagct cagacaagtc

9601 ggagcttttc cagtcgcgaa aatctgcgac tccagcggaa agcaccgaac cacagagagg

9661 actcgtatga aagccaggga agaaaccatc attcaccttg cagcaaatag gaaaaaaaaa

9721 cggacatctt caacaaacaa aagcccatgc gctaacttgg tttaggagtt tagtgtgaca

9781 ccatgacccc gctgatgatc tttacttagc accataacca cctttatgcg ttcgttcatc

9841 caaaatctac aggatatcac tgcagccgcg agaagaactc gtgaaccatc ctgttttctt

9901 ttttattata ttcttacttt taacttcaaa ttattttcag taataaaacg tctcaaaata

9961 ataagttcat aatgagttta attttacgga ataagaacaa ccatttaagt tattaaatcc

10021 ttagatttaa tggaattaga ttgattatat ggaacccaga cttggtaaaa aataaactcc

10081 acgttataat tctttctgag acttaaaatt ctttcgggaa agctgggagc aattctcgct

10141 aaggcgtcgt ccacaaatta tgaaacgctt taattacgtg acggagtagg ctcaagcgta

10201 cgaatcatac aaaaataata caaatttttc atataaaaag cgttacgaag ggggaggtgg

10261 tcgaaaattg acaattgacc ggcccaatct ttgacattag ttttctttaa taattaaaat

10321 tatgcttgat ttaaaattca tctcgagtca tctctgaatt cggttgttca attgcatggg

10381 tccttcgtta gttataatta aaaacgtgtt tgaggacgtc tcgattctaa actgttcttg

10441 gtcatggaaa atcgatgttc ttcttttatt ctctcaagat tttcaggctg tatattaaaa

10501 cttatattaa gaactatgct aaccacctca tcaggaaccg ttgtaggtgg cgtgggtttt

10561 cttggcaatc gactctcatg aaaactacga gctaaatatt caatatgttc ctcttgacca

10621 actttattct gcattttttt tgaacgaggt ttagagcaag cttcaggaaa ctgagacagg

10681 aattttatta aaaatttaaa ttttgaagaa agttcagggt taatagcatc cattttttgc

10741 tttgcaagtt cctcagcatt cttaacaaaa gacgtctctt ttgacatgtt taaagtttaa

10801 acctcctgtg tgaaattgtt atccgctcac aattccacac attatacgag ccggaagcat

10861 aaagtgtaaa gcctggggtg cctaatgagt gagctaactc acattaattg cgttgcgctc

10921 actgccaatt gctttccagt cgggaaacct gtcgtgccag ctgcattaat gaatcggcca

10981 acgcgcgggg agaggcggtt tgcgtattgg gcgctcttcc gcttcctcgc tcactgactc

11041 gctgcgctcg gtcgttcggc tgcggcgagc ggtatcagct cactcaaagg cggtaatacg

11101 gttatccaca gaatcagggg ataacgcagg aaagaacatg tgagcaaaag gccagcaaaa

11161 ggccaggaac cgtaaaaagg ccgcgttgct ggcgtttttc cataggctcc gcccccctga

11221 cgagcatcac aaaaatcgac gctcaagtca gaggtggcga aacccgacag gactataaag

11281 ataccaggcg tttccccctg gaagctccct cgtgcgctct cctgttccga ccctgccgct

11341 taccggatac ctgtccgcct ttctcccttc gggaagcgtg gcgctttctc atagctcacg

11401 ctgtaggtat ctcagttcgg tgtaggtcgt tcgctccaag ctgggctgtg tgcacgaacc

11461 ccccgttcag cccgaccgct gcgccttatc cggtaactat cgtcttgagt ccaacccggt

11521 aagacacgac ttatcgccac tggcagcagc cactggtaac aggattagca gagcgaggta

11581 tgtaggcggt gctacagagt tcttgaagtg gtggcctaac tacggctaca ctagaaggac

11641 agtatttggt atctgcgctc tgctgaagcc agttaccttc ggaaaaagag ttggtagctc

11701 ttgatccggc aaacaaacca ccgctggtag cggtggtttt tttgtttgca agcagcagat

11761 tacgcgcaga aaaaaaggat ctcaagaaga tcctttgatc ttttctacgg ggtctgacgc

11821 tcagtggaac gaaaactcac gttaagggat tttggtcatg agattatcaa aaaggatctt

11881 cacctagatc cttttaaatt aaaaatgaag ttttaaatca atc

//

LOCUS *190-recoded* 8387 bp ds-DNA circular 03-AUG-2021

DEFINITION .

FEATURES Location/Qualifiers

CDS complement(98..958)

/label="AmpR"

misc_feature complement(959..1063)

/label="AmpR Promoter"

CDS 1344..1573

/label="LacZ Alpha (Part I)"

misc_feature 1574..3699

/label="LVP 5' Hom Arm sgRNA2 SNP/100bp"

misc_feature 3700..3928

/label="3xP3"

misc_feature 3929..4629

/label="AmCyan"

3'UTR 4630..4858

/label="SV40"

misc_feature 4859..4908

/label="attP50"

misc_feature 5009..5108

/label="AeU6-3 promoter_100bp"

misc_feature 5109..5128

/label="Act4-2 (Aegyp)"

misc_RNA 5129..5204

/label="gRNA scaffold_guide RNA scaffold for the CRISPR/Cas9 system"

misc_feature 5212..5245

/label="loxP"

misc_feature 5246..7289

/label="LVP 3' Hom Arm sgRNA2 SNP/100bp"

CDS 7322..7394

/label="LacZ Alpha (Part II)"

misc_feature complement(7670..8337)

/label="pUC57 Ori (1)"

ORIGIN

1 tgagattatc aaaaaggatc ttcacctaga tccttttaaa ttaaaaatga agttttaaat

61 caatctaaag tatatatgag taaacttggt ctgacagtta ccaatgctta atcagtgagg

121 cacctatctc agcgatctgt ctatttcgtt catccatagt tgcctgactc cccgtcgtgt

181 agataactac gatacgggag ggcttaccat ctggccccag tgctgcaatg ataccgcgag

241 acccacgctc accggctcca gatttatcag caataaacca gccagccgga agggccgagc

301 gcagaagtgg tcctgcaact ttatccgcct ccatccagtc tattaattgt tgccgggaag

361 ctagagtaag tagttcgcca gttaatagtt tgcgcaacgt tgttgccatt gctacaggca

421 tcgtggtgtc acgctcgtcg tttggtatgg cttcattcag ctccggttcc caacgatcaa

481 ggcgagttac atgatccccc atgttgtgca aaaaagcggt tagctccttc ggtcctccga

541 tcgttgtcag aagtaagttg gccgcagtgt tatcactcat ggttatggca gcactgcata

601 attctcttac tgtcatgcca tccgtaagat gcttttctgt gactggtgag tactcaacca

661 agtcattctg agaatagtgt atgcggcgac cgagttgctc ttgcccggcg tcaatacggg

721 ataataccgc gccacatagc agaactttaa aagtgctcat cattggaaaa cgttcttcgg

781 ggcgaaaact ctcaaggatc ttaccgctgt tgagatccag ttcgatgtaa cccactcgtg

841 cacccaactg atcttcagca tcttttactt tcaccagcgt ttctgggtga gcaaaaacag

901 gaaggcaaaa tgccgcaaaa aagggaataa gggcgacacg gaaatgttga atactcatac

961 tcttcctttt tcaatattat tgaagcattt atcagggtta ttgtctcatg agcggataca

1021 tatttgaatg tatttagaaa aataaacaaa taggggttcc gcgcacattt ccccgaaaag

1081 tgccacctga cgtctaagaa accattatta tcatgacatt aacctataaa aataggcgta

1141 tcacgaggcc ctttcgtctc gcgcgtttcg gtgatgacgg tgaaaacctc tgacacatgc

1201 agctcccgga gacggtcaca gcttgtctgt aagcggatgc cgggagcaga caagcccgtc

1261 agggcgcgtc agcgggtgtt ggcgggtgtc ggggctggct taactatgcg gcatcagagc

1321 agattgtact gagagtgcac catatgcggt gtgaaatacc gcacagatgc gtaaggagaa

1381 aataccgcat caggcgccat tcgccattca ggctgcgcaa ctgttgggaa gggcgatcgg

1441 tgcgggcctc ttcgctatta cgccagctgg cgaaaggggg atgtgctgca aggcgattaa

1501 gttgggtaac gccagggttt tcccagtcac gacgttgtaa aacgacggcc agtgaattga

1561 cgcgtattgg gatgtcttcc gagtataaaa ccccggtaaa cccaaggaat cactcacaat

1621 cggattttga cgctcgctct ggtacagttc gatacggtct agtgaaaccg aggaaaacga

1681 cgaaggtttt tccccattga tccaggtcgg tgtttatgat tggtggaaaa agagctcgag

1741 aaaagttcca tcgaagccgt tggaaatgtg ccgtgttcct gtgacgtctt gtggatccag

1801 ttccttgttc acgtctggtg atcgtgtaaa atgtgctgtc ttgtggcgtc atatgtgttc

1861 cagatccagt gatttcgatc cgatgtgatg ttgatccctt gtgaacgtct tatcctgttc

1921 cgtgtgcacc atgcataatg tcgtattacg taagttctga agtgaaacag aagactgaat

1981 tgaaagtttt tttattcaac atcaacctaa atatggactt tactttccaa gaaaattatg

2041 cctgatcaac tgtggatagt tacaaaaaaa aaagctttat taattaaatt ttatgattac

2101 ataatgtgtt gaaaagaaca actgaaattt tagaagaaga tcttttcgtg catcaggctt

2161 tgccaattaa ttgaagataa attatcatag caaattaacg tagagactaa aaggtatatc

2221 gtcaaatagg gcttcttttg acactatttt ggcattcttg ctctttgaga acttccaacc

2281 ctaaaatggg atcttcatca gcctagtggt tagattcagc agctacaaag caaaaccatg

2341 ctgaagggtt cgattcccgg tcgtttcagg atctattcgt aattgaaata tccttgacta

2401 ccctaagtat cattgtgctt gccatttacg aatatacata ttacgatata cgaatgagaa

2461 aatgacaact ttggtaaata aagctctcaa tgtttcaata agaaataaat actacatcag

2521 tattgaaggc taataacaat tacagattag aacctttaaa catcatttct gcaagaggct

2581 ggataaagta cagttggagg attaaattat gcgattttgc aattttttcc gattaaattc

2641 atatttattc ctggtttggt ttttacaaaa aataatttta catgacgttt gaccccgatt

2701 ccctcaactt tgattgttat attttttttt ggacaggttg agtttgtggg ttttttccta

2761 gtgttgcttt gcttaatggg ctctggttat ttaaaattaa aatttgacaa tcttactaca

2821 cactccgaaa aaatcatgcg attttacgtc ttttggatgc acataaaaga agcgtgccaa

2881 atgaggtgaa tttgtgtcac attttaaata cgatggtgtc tgattcggga aatgtcaatg

2941 atagtgtcat tcaatcataa tgtgaattac gtccccagta attttcatta tttttaagag

3001 tgtactacta tttacactac aaaaattttg ataccccagg ggggaacgag gtcccggatg

3061 tccagctggc cagaatgttg gcaacgagcc ctgtacctat tgatcgagtc accaaagcac

3121 tcctcaagtg ttttaatctc gaccagacgg tggacctcgg ttgttctcat tctccgaggg

3181 cgatttcgca atcattagta ccaaccacat gtcgaagtcg ggagatgtta taaaattata

3241 accaattatt caaaaaatga catcattcaa tttgtacaaa cgttcgatag aaattatata

3301 tgatttcaca tgatattaaa ctacgaagaa aattttacat aaggaagtgg tataaaacgt

3361 aatatgctta ataataactt taaacccttt tgggaggata atattcagaa gttctgattc

3421 agaaccatct ctcatgttat gttcgttttt tgttgcttgt cctttatatg ccagatgaac

3481 aataacacca atatctatcc catttccagg acctaacgga ccttgaagcg gcgccaaaat

3541 gtgtgacgat gatgctggag cactagtcat tgacaactga tgaggcatgt gtaaggccgg

3601 tttcgctggt gatgatgccc cacgtgccgt cttcccgtcc attgtcggct gacctcgcca

3661 ccagggtgtg atggtcggta tgggtcaaaa agatgcctag cccggggatc taattcaatt

3721 agagactaat tcaattagag ctaattcaat taggatccaa gcttatcgat ttcgaaccct

3781 cgaccgccgg agtataaata gaggcgcttc gtctacggag cgacaattca attcaaacaa

3841 gcaaagtgaa cacgtcgcta agcgaaagct aagcaaataa acaagcgcag ctgaacaagc

3901 taaacaatcg gggtaccgct agagtcgaat ggccctgtcc aacaagttca tcggcgacga

3961 catgaagatg acctaccaca tggacggctg cgtgaacggc cactacttca ccgtgaaggg

4021 cgagggcagc ggcaagccct acgagggcac ccagacctcc accttcaaag tcacaatggc

4081 caacggcggc cccctggcct tctccttcga catcctgtcc accgtgttca tgtacggcaa

4141 ccgctgcttc accgcctacc ccaccagcat gcccgactac ttcaagcagg ccttccccga

4201 cggcatgtcc tacgagagaa ccttcaccta cgaggacggc ggcgtggcca ccgccagctg

4261 ggagatcagc ctgaagggca actgcttcga gcacaagtcc accttccacg gcgtgaactt

4321 ccccgccgac ggccccgtga tggccaagaa gaccaccggc tgggacccct ccttcgagaa

4381 gatgaccgtg tgcgacggca tcttgaaggg cgacgtgacc gccttcctga tgctgcaagg

4441 cggcggcaac tacagatgcc agttccacac ctcctacaag accaagaagc ccgtgaccat

4501 gccccccaac cacgtggtgg agcaccgcat cgccagaacc gacctggaca agggcggcaa

4561 cagcgtgcag ctgaccgagc acgccgtggc ccacatcacc tccgtggtgc ccttctccgg

4621 actccgctct gatcataatc agccatacca catttgtaga ggttttactt gctttaaaaa

4681 acctcccaca cctccccctg aacctgaaac ataaaatgaa tgcaattgtt gttgttaact

4741 tgtttattgc agcttataat ggttacaaat aaagcaatag catcacaaat ttcacaaata

4801 aagcattttt ttcactgcat tctagttgtg gtttgtccaa actcatcaat gtatcttaag

4861 tagtgcccca actggggtaa cctttgagtt ctctcagttg ggggcgtact ctacccgttc

4921 aggcagcatt catcgaaaag ccctatctgc tcgcacacat ttacaaaatg ctgattgcgt

4981 tgtgtgctga atgggtcact cgtccgtcac tgcttgctgt gtacactgta cagttacgca

5041 gtctgtgcat cgctagaatc atatttacgg aagagtatta tatatacccg atgcgttgct

5101 cttcgattgg tcaaaaagat gcctacgtgt tttagagcta gaaatagcaa gttaaaataa

5161 ggctagtccg ttatcaactt gaaaaagtgg caccgagtcg gtgctttttt tataacttcg

5221 tataatgtat gctatacgaa gttatcgtcg gtgatgaatg acaatccaag cgaggtatcc

5281 tctgactgaa atatcccata gagcacggta tcatcaccaa ctgggatgat atggagaaga

5341 tttggcatca caccttctac aacgagttgc gagtagctcc tgaagaacac ccagtattgc

5401 tgactgaggc tcccttgaat ccaaagtcca atcgcgagaa gatgactcag atcatgtttg

5461 aaacattcgc ttcgccagct gtgtatgtcg ccatccaagc tgttctgtcc ctgtacgcct

5521 ccggtcgtac tactggtatt gttctggatt ccggagatgg tgtctcccat accgtcccaa

5581 tctacgaggg ttatgctctg ccacatgcca tcctccgtat ggatttggct ggtcgtgatc

5641 tgaccgacta cctgatgaag atcttgaccg aacgtggata ctctttcaca accaccgctg

5701 aacgtgaaat cgttcgtgac atcaaggaga agctgtgcta cgtcgctctg gacttcgagc

5761 aggaaatgca agccgctgcc gctacgtcgt catccgagaa gtcttatgaa cttcccgatg

5821 gccaagtcat cacaatcggc aacgagcgtt tccgtgctcc agaagccctt ttccagccat

5881 ccttccttgg aatggaatca actggcattc atgaaacggt ctacaactcg atcatgcgtt

5941 gcgatgtcga catccgcaag gatctctatg ctaacagcgt cttgtctggc ggtaccacca

6001 tgtacccagg tatttcttat atttaaccac tcaactctgc atcatactca aaacctccct

6061 ctattacagg tattgctgat cgtatgcagg aggaaatcac ttccctggct ccatccacca

6121 tcaagatcaa gatcattgcc ccaccggaac gtaaatactc cgtctggatc ggtggatcca

6181 tcctggcccc gctgtctacc ttccaagcta tgtggatctc caagcaggaa tacgacgaag

6241 gtggcccagg aattgtccac cgcaagtgct tctaagccga tcccgatcgt actgattacc

6301 ataagcgaca ttgccagtga aagcgacaac agcagcatca aagtacattt gtcatactga

6361 ttcggctact accaccatcc ggaatcaggt tgcatcgaac atcaaatcac gttattcaat

6421 gtatctgtca tccagctcag acaagtcgga gcttttccag tcgcgaaaat ctgcgactcc

6481 agcggaaacc accgaaccac agagaggact cgtatgaaag ccagggaaga aaccatcatt

6541 caccttgcag caaataggaa aaaaaaacgg acatcttcaa caaacaaatg cccatgcgct

6601 aacttggttt aggagtttag tgtgacacca tgaccccgct gatgatcttt acttagcacc

6661 ataaccacct ttatgcgttc gttcatccta aatctacagg atatcactgc agccgcgaga

6721 agaactcgtg aaccatcctg ttttcttttt tattatattc ttacttttaa cttcaaatta

6781 ttttcagtta taaaacgtct caaaataata agttcataat gagtttaatt ttacggaata

6841 agaacaacca tttaagttat taaatcctta gatttaatgg aattagatag attatatgga

6901 acccagactt ggtaaaaaat aaactccacg ttaaatttct ttctgagact taaaattctt

6961 tcgggaaagc tgggagcaat tctcgctatg gagtcgtcca caaattatga aacgctttaa

7021 ttacgtaacg gagtaggctc atgcgtacga atcatacaaa aataatacaa atttttcata

7081 taacaagcct tacgaagggg gaggtggtcg aaaattgaca attgaccggc ccaatctttg

7141 acattagttt tctttaataa ttaaaattat gcttgattta aaattcatgt cgagtcatct

7201 ctgaattcgg ttgttcaatt gcatgggtcc ttcgttagtt ataattaaaa acgtgtttga

7261 ggacgtctcg attctaaact gttcttggaa tcccaatggc gcgccgagct tggcgtaatc

7321 atggtcatag ctgtttcctg tgtgaaattg ttatccgctc acaattccac acaacatacg

7381 agccggaagc ataaagtgta aagcctgggg tgcctaatga gtgagctaac tcacattaat

7441 tgcgttgcgc tcactgcccg ctttccagtc gggaaacctg tcgtgccagc tgcattaatg

7501 aatcggccaa cgcgcgggga gaggcggttt gcgtattggg cgctcttccg cttcctcgct

7561 cactgactcg ctgcgctcgg tcgttcggct gcggcgagcg gtatcagctc actcaaaggc

7621 ggtaatacgg ttatccacag aatcagggga taacgcagga aagaacatgt gagcaaaagg

7681 ccagcaaaag gccaggaacc gtaaaaaggc cgcgttgctg gcgtttttcc ataggctccg

7741 cccccctgac gagcatcaca aaaatcgacg ctcaagtcag aggtggcgaa acccgacagg

7801 actataaaga taccaggcgt ttccccctgg aagctccctc gtgcgctctc ctgttccgac

7861 cctgccgctt accggatacc tgtccgcctt tctcccttcg ggaagcgtgg cgctttctca

7921 tagctcacgc tgtaggtatc tcagttcggt gtaggtcgtt cgctccaagc tgggctgtgt

7981 gcacgaaccc cccgttcagc ccgaccgctg cgccttatcc ggtaactatc gtcttgagtc

8041 caacccggta agacacgact tatcgccact ggcagcagcc actggtaaca ggattagcag

8101 agcgaggtat gtaggcggtg ctacagagtt cttgaagtgg tggcctaact acggctacac

8161 tagaagaaca gtatttggta tctgcgctct gctgaagcca gttaccttcg gaaaaagagt

8221 tggtagctct tgatccggca aacaaaccac cgctggtagc ggtggttttt ttgtttgcaa

8281 gcagcagatt acgcgcagaa aaaaaggatc tcaagaagat cctttgatct tttctacggg

8341 gtctgacgct cagtggaacg aaaactcacg ttaagggatt ttggtca

//

LOCUS *234-recoded* 8387 bp ds-DNA circular 03-AUG-2021

DEFINITION .

FEATURES Location/Qualifiers

CDS complement(86..946)

/label="AmpR"

misc_feature complement(947..1051)

/label="AmpR promoter-006_"

CDS 1332..1561

/label="LacZ Alpha (Part I)"

misc_feature 1562..3731

/label="LVP 5' Hom Arm sgRNA3 SNP/100bp"

misc_feature 3732..3960

/label="3xP3"

misc_feature 3961..4661

/label="AmCyan"

3'UTR 4662..4890

/label="SV40"

misc_feature 4891..4940

/label="attP50"

misc_feature 5041..5140

/label="AeU6-3 promoter_100bp"

misc_feature 5141..5160

/label="Act4-3 (Aegyp)"

misc_RNA 5161..5236

/label="gRNA scaffold_guide RNA scaffold for the CRISPR/Cas9 system"

misc_feature 5244..5277

/label="loxP"

misc_feature 5278..7277

/label="LVP 3' Hom Arm sgRNA3 SNP/100bp"

CDS 7310..7382

/label="LacZ Alpha (Part II)"

misc_feature complement(7658..8325)

/label="pUC57 Ori (1)"

ORIGIN

1 aaaggatctt cacctagatc cttttaaatt aaaaatgaag ttttaaatca atctaaagta

61 tatatgagta aacttggtct gacagttacc aatgcttaat cagtgaggca cctatctcag

121 cgatctgtct atttcgttca tccatagttg cctgactccc cgtcgtgtag ataactacga

181 tacgggaggg cttaccatct ggccccagtg ctgcaatgat accgcgagac ccacgctcac

241 cggctccaga tttatcagca ataaaccagc cagccggaag ggccgagcgc agaagtggtc

301 ctgcaacttt atccgcctcc atccagtcta ttaattgttg ccgggaagct agagtaagta

361 gttcgccagt taatagtttg cgcaacgttg ttgccattgc tacaggcatc gtggtgtcac

421 gctcgtcgtt tggtatggct tcattcagct ccggttccca acgatcaagg cgagttacat

481 gatcccccat gttgtgcaaa aaagcggtta gctccttcgg tcctccgatc gttgtcagaa

541 gtaagttggc cgcagtgtta tcactcatgg ttatggcagc actgcataat tctcttactg

601 tcatgccatc cgtaagatgc ttttctgtga ctggtgagta ctcaaccaag tcattctgag

661 aatagtgtat gcggcgaccg agttgctctt gcccggcgtc aatacgggat aataccgcgc

721 cacatagcag aactttaaaa gtgctcatca ttggaaaacg ttcttcgggg cgaaaactct

781 caaggatctt accgctgttg agatccagtt cgatgtaacc cactcgtgca cccaactgat

841 cttcagcatc ttttactttc accagcgttt ctgggtgagc aaaaacagga aggcaaaatg

901 ccgcaaaaaa gggaataagg gcgacacgga aatgttgaat actcatactc ttcctttttc

961 aatattattg aagcatttat cagggttatt gtctcatgag cggatacata tttgaatgta

1021 tttagaaaaa taaacaaata ggggttccgc gcacatttcc ccgaaaagtg ccacctgacg

1081 tctaagaaac cattattatc atgacattaa cctataaaaa taggcgtatc acgaggccct

1141 ttcgtctcgc gcgtttcggt gatgacggtg aaaacctctg acacatgcag ctcccggaga

1201 cggtcacagc ttgtctgtaa gcggatgccg ggagcagaca agcccgtcag ggcgcgtcag

1261 cgggtgttgg cgggtgtcgg ggctggctta actatgcggc atcagagcag attgtactga

1321 gagtgcacca tatgcggtgt gaaataccgc acagatgcgt aaggagaaaa taccgcatca

1381 ggcgccattc gccattcagg ctgcgcaact gttgggaagg gcgatcggtg cgggcctctt

1441 cgctattacg ccagctggcg aaagggggat gtgctgcaag gcgattaagt tgggtaacgc

1501 cagggttttc ccagtcacga cgttgtaaaa cgacggccag tgaattgacg cgtattggga

1561 tgacttccga gtataaaacc ccggtaaacc caaggaatca ctcacaatcg gtttttgacg

1621 ctcgctctgg tacagttcga tacggtctag tgaaaccgag gataacgacg aaggtttttc

1681 cccattgatc caggtcggtg tttatgattg gaggaaaaag agctcgagaa aagttccatc

1741 gaagccgttg gaaatgtgcc gtcttcctgt gacgtcttgt ggatccagtt ccttgttcac

1801 gtctggtgat cctgtaaaat gtgctgtctt gtggcgtcat atgtgttcca gatccagtga

1861 ttacgatccg atgtgatgtt gatcccttgt gaacgtctta tcctgttccg tctgcaccat

1921 gcataatgtc gtattacgta agttctgaag tgaaacagaa gagtgaattg aaagtttttt

1981 tattcaacat caacctaaat atggacttta cattccaaga aaattatgcc tgatcaactg

2041 tggatagtta caaaaaaaaa aggtttatta attaaatttt atgattacat aatgtgttga

2101 aaagaacaac tcaaatttta gaagaagatc ttttcgtgca tcaggctttg ccaattaatt

2161 gatgataaat tatcatagca aattaacgta gagactaaaa ggtatatcgt ctaatagggc

2221 ttcttttgac actattttgg cattcttgct ctttgagaac ttgcaaccct aaaatgggat

2281 cttcatcagc ctagtggtta gattcagcag caacaaagca aaaccatgct gaagggttcg

2341 attcccggtc gtttcaggat cttttcgtaa ttgaaatatc cttgactacc ctaagtatca

2401 ttgtgcttgc cttttacgaa tatacatatt acgatatacg aatgagaaaa tgacaacttt

2461 ggaaaataaa gctctcaatg tttcaataag aaataaatac tacatcagta tagaaggcta

2521 ataacaatta cagattagaa cctttaaaca tcatttctgc aacaggctgg ataaagtaca

2581 gttggaggat taaattatgc gattttgcaa tattttccga ttaaattcat atttattcct

2641 ggtttggttt ttacaaaaaa tatttttaca tgacgtttga ccccgattcc ctcaactttg

2701 attgttatat tattttttgg acaggttgag tttgtgggtt ttttcctagt gttgctttgc

2761 tttatgggct ctggttattt aaaattaaaa tttgacaatc ttactacaca caccgaaaaa

2821 atcatgcgat tttacgtctt ttggatgcac ataaaagaag cgagccaaat gaggtgaatt

2881 tgtgtcacat tttaaatacg atggtgtctg aatcgggaaa tgtcaatgat agtgtcattc

2941 aatcataatg tgaattacgt ccgcagtaat tttcattatt tttaagagtg tactactatt

3001 tacactacaa atattttgat accccagggg ggaacgaggt cccggatgtc cagctggcca

3061 gattgttggc aacgagccct gtacctattg atcgagtcac caaagcactc cacaagtgtt

3121 ttaatctcga ccagacggtg gacctcggtt gttctcattc tcggagggcg atttcgcaat

3181 cattagtacc aaccacatgt cgaagtcggg acatgttata aaattataac caattattca

3241 aaaaatgaca tcattcaatt tgaacaaacg ttcgatagaa attatatatg atttcacatg

3301 atattaaact aggaagaaaa ttttacataa ggaagtggta taaaacgtaa tatgcttaat

3361 aaaaacttta aacccttttg ggaggataat attcagaagt tctgattcag atccatctct

3421 catgttatgt tcgttttttg ttgcttgtcc tttatatgcc acatgaacaa taacaccaat

3481 atctatccca tttccaggac ctaacggacc tagaagcggc gccaaaatgt gtgacgatga

3541 tgctggagca ctagtcattg acaactaata aggcatgtgt aaggccggtt tcgctggtga

3601 tgatgcccca cgtgccgtct tcccgtccat tgtcggctaa cctcgccacc agggtgtgat

3661 ggtcggtatg ggtcaaaaag atgcctacgt ctaagatgaa taacaatcca agcgaggtat

3721 cctcaccctg agcccgggga tctaattcaa ttagagacta attcaattag agctaattca

3781 attaggatcc aagcttatcg atttcgaacc ctcgaccgcc ggagtataaa tagaggcgct

3841 tcgtctacgg agcgacaatt caattcaaac aagcaaagtg aacacgtcgc taagcgaaag

3901 ctaagcaaat aaacaagcgc agctgaacaa gctaaacaat cggggtaccg ctagagtcga

3961 atggccctgt ccaacaagtt catcggcgac gacatgaaga tgacctacca catggacggc

4021 tgcgtgaacg gccactactt caccgtgaag ggcgagggca gcggcaagcc ctacgagggc

4081 acccagacct ccaccttcaa agtcacaatg gccaacggcg gccccctggc cttctccttc

4141 gacatcctgt ccaccgtgtt catgtacggc aaccgctgct tcaccgccta ccccaccagc

4201 atgcccgact acttcaagca ggccttcccc gacggcatgt cctacgagag aaccttcacc

4261 tacgaggacg gcggcgtggc caccgccagc tgggagatca gcctgaaggg caactgcttc

4321 gagcacaagt ccaccttcca cggcgtgaac ttccccgccg acggccccgt gatggccaag

4381 aagaccaccg gctgggaccc ctccttcgag aagatgaccg tgtgcgacgg catcttgaag

4441 ggcgacgtga ccgccttcct gatgctgcaa ggcggcggca actacagatg ccagttccac

4501 acctcctaca agaccaagaa gcccgtgacc atgcccccca accacgtggt ggagcaccgc

4561 atcgccagaa ccgacctgga caagggcggc aacagcgtgc agctgaccga gcacgccgtg

4621 gcccacatca cctccgtggt gcccttctcc ggactccgct ctgatcataa tcagccatac

4681 cacatttgta gaggttttac ttgctttaaa aaacctccca cacctccccc tgaacctgaa

4741 acataaaatg aatgcaattg ttgttgttaa cttgtttatt gcagcttata atggttacaa

4801 ataaagcaat agcatcacaa atttcacaaa taaagcattt ttttcactgc attctagttg

4861 tggtttgtcc aaactcatca atgtatctta agtagtgccc caactggggt aacctttgag

4921 ttctctcagt tgggggcgta ctctacccgt tcaggcagca ttcatcgaaa agccctatct

4981 gctcgcacac atttacaaaa tgctgattgc gttgtgtgct gaatgggtca ctcgtccgtc

5041 actgcttgct gtgtacactg tacagttacg cagtctgtgc atcgctagaa tcatatttac

5101 ggaagagtat tatatatacc cgatgcgttg ctcttcgatt gtgctctatg ggatatttca

5161 gttttagagc tagaaatagc aagttaaaat aaggctagtc cgttatcaac ttgaaaaagt

5221 ggcaccgagt cggtgctttt tttataactt cgtataatgt atgctatacg aagttataat

5281 atcccataga gcacggtatc atcaccaact gggatgatat ggagaaaatt tggcatcaca

5341 ccttctacaa cgagttgcga gtagctcctg aagaacatcc agtattgctg actgaggctc

5401 ccttgaatcc aaagtccaat cgcgaaaaga tgactcagat catgtttgaa acattcgctt

5461 cgccagctgt gtatgttgcc atccaagctg ttctgtccct gtacgcctcc ggtcgtacta

5521 ctggtatcgt tctggattcc ggagatggtg tctcccatac cgtcccaatc tacgaaggtt

5581 atgctctgcc acatgccatc ctccgtatgg atttggctgg tcgtgacctg accgactacc

5641 tgatgaagat cttgaccgaa cgtggatact ctttcaccac caccgctgaa cgtgaaatcg

5701 ttcgtgacat caaggagaag ctgtgttacg tcgctctgga cttcgagcag gaaatgcaag

5761 ccgctgccgc tacgtcttca tccgagaagt cttatgaact tcccgatggc caagtcatca

5821 caatcggaaa cgagcgtttc cgtgctccag aagccctttt ccagccatcc ttcctgggaa

5881 tggaatcaac tggcattcat gaaacggtct acaactcgat catgcgctgc gatgtcgaca

5941 tccgcaagga tctctatgct aacagcgtct tgtctggtgg taccaccatg tacccaggta

6001 tttcttatat ttaaccactc aactctccat catactcaaa acctccctct attacaggta

6061 ttgctgatcg tatgcagaag gaaatcactt ccctggctcc atccaccatc aagatcaaga

6121 tcattacccc accggaacgt aaatactccg tctggatcgg tggatccatc ctggcctcgc

6181 tgtctacctt ccaagctatg tggatctcca agcaggaata cgacgaaagt ggcccaggaa

6241 ttgtccaccg caagtgcttc taagccgatc ccgattgtac tgattaccat aagcgacatt

6301 gccagtgaaa gcgacaacag cagcattaaa gtacatttgt catactgatt cggctactac

6361 caccatccgg aatcagcttg catcgaacat caaatcacgt tattcaatgt atctgtcatc

6421 cagctcaggc aagtcggagc ttttccagtc gcgaaaatct gcgactccag cggaaagcac

6481 cgaaccacag agaggactcg tatgaaagcc agggaagaaa ccatcatcca ccttgcagca

6541 aataggaaaa aaaaacggac atcttcaaca aacaaaagcc catgcgctaa cttggtttag

6601 gagtttagtg tgacaccatg accccggtga tgatctttac ttagcaccat aaccaccttt

6661 atgcgttcgt tcatccaaaa tctacaggat atcactgcag ccgcgagaag aactcgtgaa

6721 ccatcctgtt ttctttttta ttatattctt acttttaact tcaaattatt ttcagtaata

6781 aaacgtctca aaataataag ttcataatga gtttaatttt acggaaaaag aacaaccatt

6841 taagttatta aatccttaga tttaatggaa ttagattgat tatatggaac ccagacttgg

6901 taaaaaataa actccacgtt aaatttgttt ctgagactta aaattctttc gggaaagctg

6961 ggagcaattc tcgctaagga gtcgtccaca aattatgaaa cgctttaatt acgtaacgga

7021 gtaggcacat gcgtacgaat catacaaaaa taatacaaat ttttcatata acaagcgtta

7081 cgaaggggga ggtggtcgaa aattgacaat tgaccggccc aatcttagac attagttttc

7141 tttaataatt aaaattatgc ttgatttaaa attcatctcg agtcatctct gaattcggtt

7201 gttcaattgc atgggtcctt cgttagatat aattaaaaac gtgtttgagg acgtctcgat

7261 tctaaactgt tcttggtatc ccaatggcgc gccgagcttg gcgtaatcat ggtcatagct

7321 gtttcctgtg tgaaattgtt atccgctcac aattccacac aacatacgag ccggaagcat

7381 aaagtgtaaa gcctggggtg cctaatgagt gagctaactc acattaattg cgttgcgctc

7441 actgcccgct ttccagtcgg gaaacctgtc gtgccagctg cattaatgaa tcggccaacg

7501 cgcggggaga ggcggtttgc gtattgggcg ctcttccgct tcctcgctca ctgactcgct

7561 gcgctcggtc gttcggctgc ggcgagcggt atcagctcac tcaaaggcgg taatacggtt

7621 atccacagaa tcaggggata acgcaggaaa gaacatgtga gcaaaaggcc agcaaaaggc

7681 caggaaccgt aaaaaggccg cgttgctggc gtttttccat aggctccgcc cccctgacga

7741 gcatcacaaa aatcgacgct caagtcagag gtggcgaaac ccgacaggac tataaagata

7801 ccaggcgttt ccccctggaa gctccctcgt gcgctctcct gttccgaccc tgccgcttac

7861 cggatacctg tccgcctttc tcccttcggg aagcgtggcg ctttctcata gctcacgctg

7921 taggtatctc agttcggtgt aggtcgttcg ctccaagctg ggctgtgtgc acgaaccccc

7981 cgttcagccc gaccgctgcg ccttatccgg taactatcgt cttgagtcca acccggtaag

8041 acacgactta tcgccactgg cagcagccac tggtaacagg attagcagag cgaggtatgt

8101 aggcggtgct acagagttct tgaagtggtg gcctaactac ggctacacta gaagaacagt

8161 atttggtatc tgcgctctgc tgaagccagt taccttcgga aaaagagttg gtagctcttg

8221 atccggcaaa caaaccaccg ctggtagcgg tggttttttt gtttgcaagc agcagattac

8281 gcgcagaaaa aaaggatctc aagaagatcc tttgatcttt tctacggggt ctgacgctca

8341 gtggaacgaa aactcacgtt aagggatttt ggtcatgaga ttatcaa

//

>Act4_v2_sgRNAs

ccgcggtgcgggtgccagggcgtgcccttgggctccccgggcgcgtactccgccggccagttgggacgtttgactttttgtaggtagacaaaaactaaactttttttcgcttctctatgtgtgcccccccgggtagcgtatcgttccgattgtggtgcgaacgaatgaaatcgcccatcgagttgatacgtccatccatcgctagaaccgcgttcgctgtagaagactatataagagcagaggcaagagtagtgaaatggagcactagtcattgacaagttttagagctagaaatagcaagttaaaataaggctagtccgttatcaacttgaaaaagtggcaccgagtcggtgctttttttatgctccattctccgccacttgttgatgcggaccctaaccacgtggtcgctcctctgctcaccggagcacgtttcatacagcctgacgacgacgagcaatcagaggtatggtgagcatgcgcatggagagtggacagcagtgcaccctaaaatcaattcacacatcatgtgtcaatagctgtgtcaatgttgcacagccttttcttattaaatttactccttttgtgaccatttctctttcatccaccgttattttaatgagttttgtgttccggtggacgaacgttcacacaaaaaatgtgtaaatcttaatcaaccagaacacaaagtatagtgaaaaaattaaagtgtgtggcttttatacatcctaactgtaaattatttttagagtgcgtgcgatcgttctctcgaaccacgctctccgctacacattcgcagcgaatggcgtgaatggatgaaagaacaaactaaagtttatttttagattcgtctcaaaacaactgctgtgcatcgctagaaccaagaaatacgccactcagtatatatagcacttccaaccccgctttcctcggtcaaaaagatgcctacgtgttcgagaggacgagagtccaagttcgaataaggccagtccgttatcacagggagacctgggcaccgagtcggtgctttttttctctacccgttcaggcagcattcatcgaaaagccctatctgctcgcacacatttacaaaatgctgattgcgttgtgtgctgaatgggtcactcgtccgtcactgcttgctgtgtacactgtacagttacgcagtctgtgcatcgctagaatcatatttacggaagagtattatatatacccgatgcgttgctcttcgattgtgctctatgggatatttcagttccagagtcgggaacgacaagttggaataaggcaagtccgttatcatgccggaaggcaggcaccgattcggtgcttttttttagcatagtcagtcagtgaataagcacccaagccaagcgaccatccaccgaacaactgaggagggagattgttacaagcacagctgattcgatctcgctctcggtaccgcatggcttgcgaggcaagagcatcaagctacgagcaggcaaaacaacacccctcataaaccgtaaacatacccttacgcttaccccatccattccctaccgtagcgaccagctcattatgggaaacccgaaacagattttatgttatgcttctttctctgccacttgttgatgccgcatcaagagaccacgcggtcgctcgaccacgttccatgcggcacaggtagaggaggtaggcaatcgagtgcgcgccagaggcatggtgagcatgcgcacagagagacgaccgcaccatgtgaggcgatcgttctctccgttctgaaagctctcctcgctccgtcgttttgaattaatttgtatgagtaaaggtaggcaaaagttatttttagccactcgactcgagacgttgaattcatagcaactgccatccatcgctaaaaccgaaattttcgcagtctactatatatacgacttccaccaccggatatcttctcacaccctggtggcgaggggtgttagagggatagagatatcccaagttaacataaggctagtccgttatcactgcaggaatgcagggcaccgagtcggtgctttttttgccgcgggtcgacaagctttacgagcatatg

>Add_HA

tcattgacaacggatccggcatgtgtaaggccggtttcgctggtgatgatgccccacgtgccgtcttcccgtccattgtcggccgccctcgccaccagggtgtgatggtcggtatgggtcaaaaagatgccta

>U6_sgRNA2_Add_HA

agtagtgccccaactggggtaacctttgagttctctcagttgggggcgtactctacccgttcaggcagcattcatcgaaaagccctatctgctcgcacacatttacaaaatgctgattgcgttgtgtgctgaatgggtcactcgtccgtcactgcttgctgtgtacactgtacagttacgcagtctgtgcatcgctagaatcatatttacggaagagtattatatatacccgatgcgttgctcttcgattggtcaaaaagatgcctacgtgttttagagctagaaatagcaagttaaaataaggctagtccgttatcaacttgaaaaagtggcaccgagtcggtgctttttttataacttcgtataatgtatgctatacgaagttatcgtcggtgatgaagcccaatccaagcgaggtatcctcaccctga


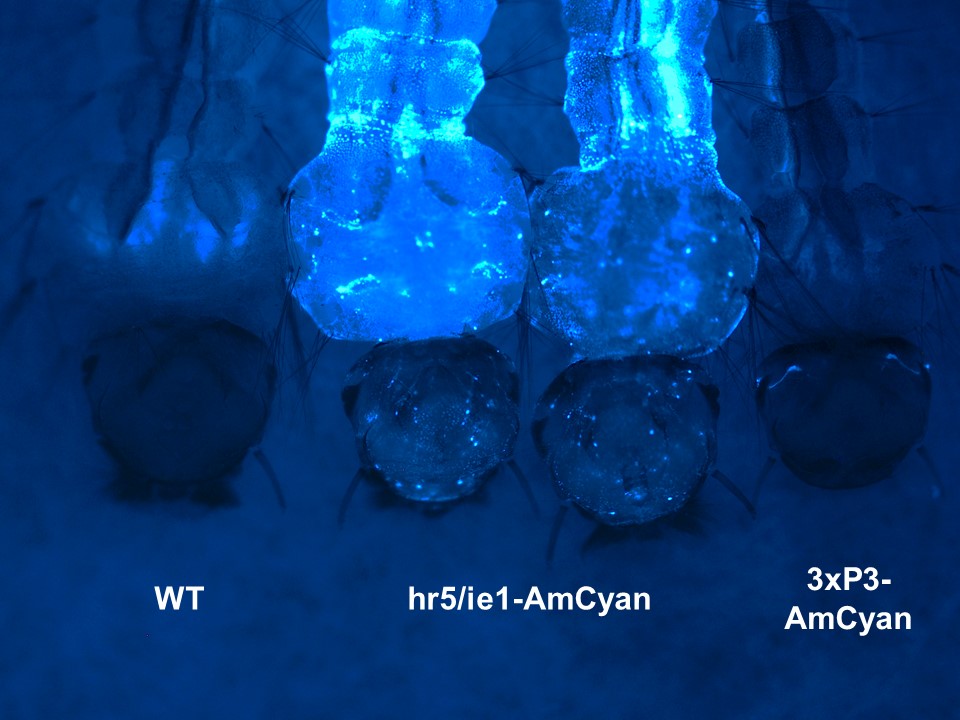
Fig A. Fluorescence patterns of hr5/ie1-AmCyan (*64+234-perfect* and *190-perfect*) and the eye-specific 3xP3-AmCyan (*190-recoded* and *234-recoded*) with canonical integrations (as determined by PCR) into the *Act4* locus.


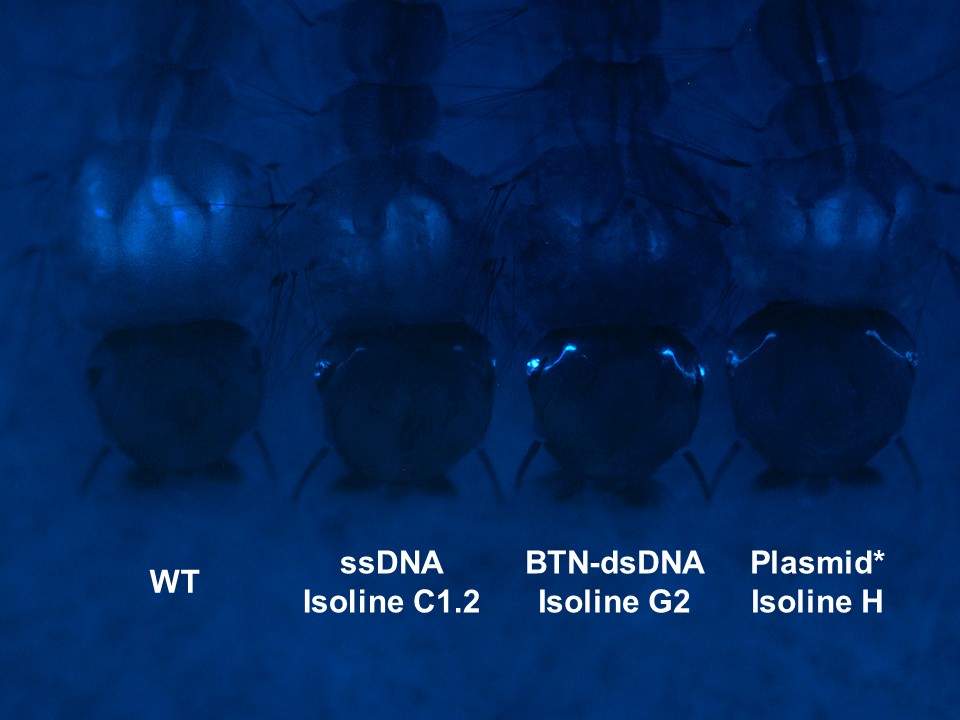
Fig B. Different fluorescence intensity of 3xP3-AmCyan expression observed in different isolines. Isoline H is a canonical integration generated with plasmid *234-recoded* while the isolines C1.2 and G2 are off-target integrations generated with ssDNA and BTN-dsDNA donors of *190-recoded*. Isoline G2 gives consistently stronger expression of the 3xP3-AmCyan marker than H, while C1.2 is weaker.

References

1. Navarro-Payá D, Flis I, Anderson MAE, Hawes P, Li M, Akbari OS, et al. Targeting female flight for genetic control of mosquitoes. PLoS Negl Trop Dis. 2020. doi:10.1371/journal.pntd.0008876
