# Supplementary material for: Considerations for homology-based DNA repair in mosquitoes: Impact of sequence heterology and donor template source
Source: PLoS Genet. 2022 Feb 18;18(2):e1010060. doi: 10.1371/journal.pgen.1010060 (PMC8893643; doi:10.1371/journal.pgen.1010060)
Supplement: S2 File — (DOCX) [file pgen.1010060.s002.docx]

## S2 File. Statistical analysis of pool positivity

As described in our methods, we have pooled injected G_0_ survivors and screened for integration events in each pool. Any pool giving rise to one or more positive G_1_ larvae is classified as positive. Here we perform a simple binomial probability analysis to account for the pooling of G_0_ survivors and estimate the underlying individual G_0_ integration rate and the statistical significance of differences observed. We designate $p$ as the probability that an individual G_0_ will give rise to positive progeny and $1-p$ as the probability that the individual G_0_ does not produce any positive progeny. From that, we can calculate the likelihood that a pool of ‘N’ G_0_ adults produce at least one positive progeny $1-((1-p)^{N})$. For a given value of $p$, the probability of the 13/13 positive pools we observed is:

$P(13/13)=\prod_{i=1}^{13} 1-(1-p)^{N_{i}}$,

$$N=\{22,22,22,22,27,18,20,20,20,20,20,20,18\}$$

For negative pools, we calculate the probability of getting exactly zero positive progeny for ‘M’ G_0_ adults. $(1-p)^{M}$ We perform the same analysis for the other groups:

$P(3/9)=\prod_{i=1}^{3} 1-(1-p)^{N_{i}}\cdot\prod_{j=1}^{6} (1-p)^{M_{j}}$,
$N=\{20,20,21\}$,

$$M=\{20,20,20,21,21,21\}$$

$$P(8/16)=\prod_{i=1}^{8} 1-(1-p)^{N_{i}}\cdot\prod_{j=1}^{8} (1-p)^{M_{j}}$$

,
$N=\{20,20,24,20,20,20,26,22\}$,

$$M=\{20,20,20,20,25,22,20,20\}$$

$$P(8/17)=\prod_{i=1}^{8} 1-(1-p)^{N_{i}}\cdot\prod_{j=1}^{9} (1-p)^{M_{j}}$$

,
$N=\{20,20,20,20,20,20,20,28\}$,

$$M=\{20,23,20,20,20,22,22,20,20\}$$

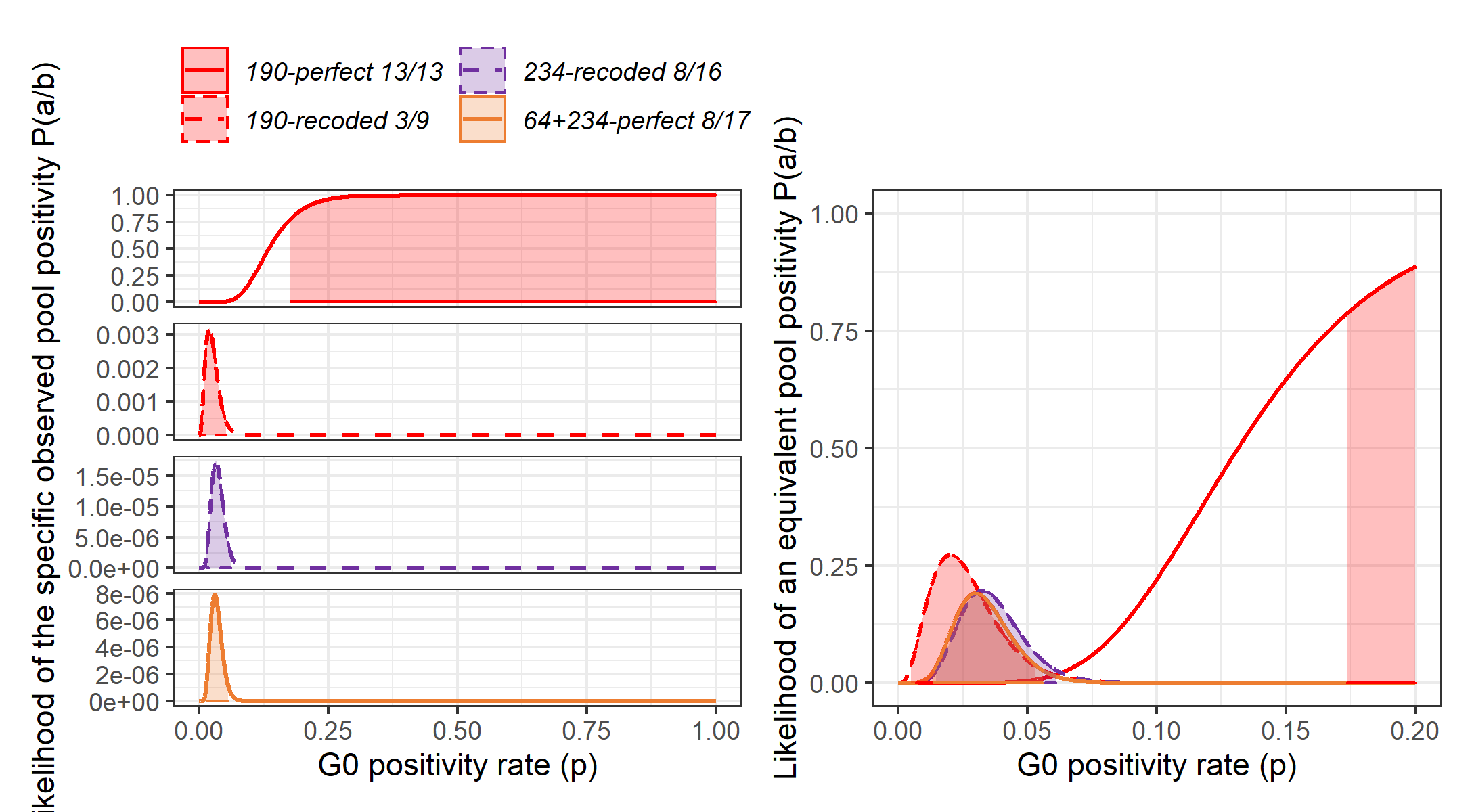
Figure S2A. Probability of the observed pool positivity (y-axis) for different individual G_0_ positivity rates (x-axis). (left) Probability of exactly the combination/order of positive pools observed. (right). Probability of equivalent combinations/orders giving the same overall pool positivity. The height of each line indicates the probability of getting that outcome given the G_0_ positivity rate. For each figure, the shaded area indicates 95% of the area occupied by each line and corresponds to the range of G_0_ positivity rates most likely to give the outcome the lines represent. Data for the right figure was calculated using the ‘dbinom()’ function in R. For the left graph, the exact pool sizes were used, while for the graph on the right, the average pool size rounded to the nearest integer was used for each group (20 for *190-recoded*, 21 for the other three groups). Analysis performed at lower resolution without taking the average pool size gave equivalent outcomes. The code for both approaches is included.

The outcome of this calculation for each group is shown on the left side of Figure S1A. For each group, the most likely value of the individual G_0_ positivity rate (p) is: 100 (95% interval:17.6-100) for *190-perfect*, 1.96 (95% interval:0.47-5.16) for *190-recoded*, 3.24 (95% interval:1.46-6.09) for *234-recoded*, and 3.01 (95% interval:1.36-5.64) for *64+234-perfect*. Note that the height of each line on the left side of Figure S2A corresponds to the likelihood of observing the specific positive and negative pool combination. This gives the best estimate of the G_0_ integration rate but does not reflect the additional specific order of the positive and negative pools that would give the same overall outcome. We extended this analysis to include the likelihood of observing any other combination/order of negative and positive pools that give the same overall outcome. This is shown on the right side of Figure S2A. Each line corresponds to a specific outcome, with the height being the likelihood of that outcome given the particular G_0_ integration rate. Both approaches show that there is substantial overlap in the individual G_0_ integration rates that would give rise to each of the other observed pool positivity rates. However, no G_0_ integration rates are likely to give rise to both 13/13 positive pools and any of the other observed pool positivity rates.
